# Oxygen Vacancy‐Enriched Platinum Single‐Atom Nanozyme Wrapped in Nanoislands: Unlocking Catalytic Activity and Reprogramming Redox Microenvironment for Osteonecrosis Repair

**DOI:** 10.1002/advs.75840

**Published:** 2026-05-26

**Authors:** Yang Zhu, Zehui Lv, Xuejie Cai, Penghui Wei, Dengliang Wang, Zhao Wang, Ruoying Wang, Yingjie Wang, Xingdong Yang, Yixin Bian, Jiawei Xu, Xisheng Weng, Liangfeng Wei

**Affiliations:** ^1^ Department of Neurosurgery, Neurosurgery Research Institute The First Affiliated Hospital Fujian Medical University Fuzhou Fujian China; ^2^ Department of Neurosurgery, National Regional Medical Center Binhai Campus of the First Affiliated Hospital Fujian Medical University Fuzhou Fujian China; ^3^ Department of Orthopedic Surgery, State Key Laboratory of Complex Severe and Rare Diseases Peking Union Medical College Hospital Chinese Academy of Medical Science and Peking Union Medical College Beijing China; ^4^ Department of Neurosurgery Fuzong Clinical Medical College of Fujian Medical University Fuzhou Fujian China

**Keywords:** cerium dioxide, nanoislands, osteonecrosis, oxygen vacancies, single atom nanozyme

## Abstract

Excessive accumulation of reactive oxygen species (ROS) impairs bone regeneration and angiogenesis in steroid‐induced osteonecrosis of the femoral head (SONFH), yet current antioxidant therapies remain limited by low catalytic efficiency and short duration. Single‐atom nanozymes (SANs), with their well‐defined structures and maximal atomic efficiency, show great potential for treating ROS‐induced diseases by mimicking natural enzymes. However, the strong binding between transition metal sites and electron‐donating intermediates (e.g., O*, OH*, OOH*) creates high energy barriers, limiting their catalytic activities. Herein, single‐atomic platinum is successfully embedded into CeO_2‐x_ to form CeO_2‐x_/Pt SANI, which enhanced catalytic activity via an “island‐sea” synergistic effect. Leveraging the unique charge‐transfer structures and confinement effect of nanoislands, CeO_2‐x_/Pt SANI exhibits superior enzymatic activities than CeO_2_, attribute to the island‐sea synergistic effect that facilitates strong electron transfer, as proved by density functional theory (DFT) calculations. DFT calculations further demonstrate that Pt incorporation increases oxygen vacancies and tunes the d‐band center toward the Fermi level, facilitating ROS adsorption and accelerating redox reactions. Single‐cell sequencing and experimental results confirm that CeO_2‐x_/Pt SANI reprograms the oxidative microenvironment, leading to significant therapeutic effects in SONFH. This study provides insights into the rational design of an advanced “island‐sea” structured single‐atom nanozyme to optimize the catalytic activity.

## Graphical abstract

CeO_2‐x_/Pt single‐atom nano‐island is constructed via low‐temperature reduction, featuring abundant oxygen vacancies and an island–sea structure. It delivers outstanding multi‐enzyme‐mimetic activity and ROS‐scavenging efficiency. Density functional theory reveals optimized electronic structures and reaction pathways. This nanozyme remodels the redox microenvironment in SONFH, restores mitochondrial function, and promotes osteogenesis and angiogenesis, opening a new catalytic route for osteonecrosis therapy.

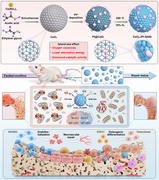

## 1 Introduction

Steroid‐induced osteonecrosis of the femoral head (SONFH) is a debilitating orthopedic disorder characterized by progressive bone necrosis and structural collapse of the femoral head [1, 2, 3]. Clinically, prolonged or high‐dose glucocorticoid (GC) administration represents a major risk factor, accounting for a substantial proportion of non‐traumatic osteonecrosis cases, though less than half [4, 5]. Despite its clinical relevance, the underlying pathogenesis remains incompletely defined [6, 7]. A growing body of evidence implicates oxidative stress as a central mediator [8]. Sustained GC exposure leads to excessive production of reactive oxygen species (ROS), disrupting redox homeostasis in bone cells and triggering mitochondrial dysfunction, cellular senescence and apoptosis [9]. In human bone marrow mesenchymal stem cells (HBMSCs), ROS accumulation impairs osteogenic differentiation, promotes adipogenic lineage commitment and accelerates cell death‐ultimately compromising bone regeneration [10, 11]. Concurrently, oxidative stress contributes to a pseudo‐hypoxic microenvironment, disrupting angiogenic signaling and leading to microvascular degeneration in the femoral head [12]. Together, these events form a pathological cascade of oxidative injury, impaired tissue repair and compromised vascular supply. Thus, there is an urgent and unmet need to develop novel antioxidant agents with broad‐spectrum catalytic activity and alleviate vascular impairment for effective and mitigate SONFH progression.

Nanozymes, artificial nanomaterials endowed with enzyme‐like activities, have attracted significant attention for their exceptional stability, cost‐effectiveness and tunable catalytic performance [13, 14, 15, 16, 17]. For instance, cerium oxide (CeO_2_) nanozyme exhibits multiple catalytic properties, such as hydroxyl radicals (•OH) scavenging, superoxide dismutase (SOD), peroxidase (POD) and catalase (CAT), which attributed to the Ce^3+^/Ce^4+^ redox pair structure [18, 19, 20]. Defect engineering, particularly the deliberate introduction of oxygen vacancies, has emerged as a remarkably effective and straightforward strategy to optimize or amplify the catalytic activity of nanozymes [21, 22]. However, the catalytic efficacy of CeO_2_ is fundamentally limited by its low density of oxygen vacancies, which restricts the adsorption and turnover of electron‐donating intermediates (e.g., O*, OH*, OOH*) [23]. Recently, single‐atom nanozymes (SANs) has garnered substantial attention in the biomedical field owing to their well‐defined electronic and geometric structures, superior substrate selectivity and maximal atomic utilization efficiency [24, 25, 26, 27, 28]. Among these, nitrogen‐doped carbon‐anchored transition metal‐based nanozymes with metal‐N_x_ active sites have been extensively investigated [29, 30, 31, 32, 33, 34]. However, the tendency of SANs to aggregate and form larger atomic clusters or nanoparticles often results in diminished enzyme‐like activity or even total inactivation [35, 36, 37, 38, 39]. On the other hand, the excessively strong binding between transition metal single‐atom sites and electron‐donating intermediates leads to high energy barriers for the catalytic reaction intermediates, severely impeding the catalytic activity of SANs [40, 41, 42, 43]. Consequently, developing novel SANs with board‐spectrum ROS elimination and lower energy barriers is crucial for the practical management of SONFH.

To tackle this challenge, the innovative concept of nanoislands has been introduced, ingeniously designed to bolster the durability and activity of single‐atom catalysts by allowing metal atoms to migrate within designated nanoisland domains while effectively restricting them to their assigned regions. For example, the formation of oxygen vacancies at the noble metal‐support interface, coupled with lattice distortion induced by noble metal embedding, profoundly alters the electronic properties of SANs, thereby modulating the adsorption and dissociation of substrate molecules and significantly enhancing their enzymatic activity through the synergistic interplay between the confined islands and the surrounding matrix. These systems are ingeniously designed with distinct “islands” (active sites dispersed on small functional carriers) and “seas” (high‐surface‐area supports), each fulfilling specific roles in catalytic reactions to effectively address multifaceted challenges. In CAT‐ and SOD‐like reactions, nano‐islands are ingeniously designed to serve as anchors for atomically dispersed metal atoms, ensuring stable and selective catalysis, while the support, acting as a “sea”, provides a neutral microenvironment conducive to the reaction. Importantly, CeO_2‐x_ nano‐islands containing oxygen vacancies can be serve as a scaffold to guide the deposition of noble single atoms, significantly optimizing energy barriers and boosting catalytic performances. Therefore, embedding noble metal single atoms into CeO_2‐x_ nanoisland supports and creating oxygen vacancies at the metal‐nanoisland interface is anticipated to markedly enhance enzymatic activity for ROS detoxification, with significant potential for treating oxidative stress‐related conditions such as SONFH.

**FIGURE 1:**
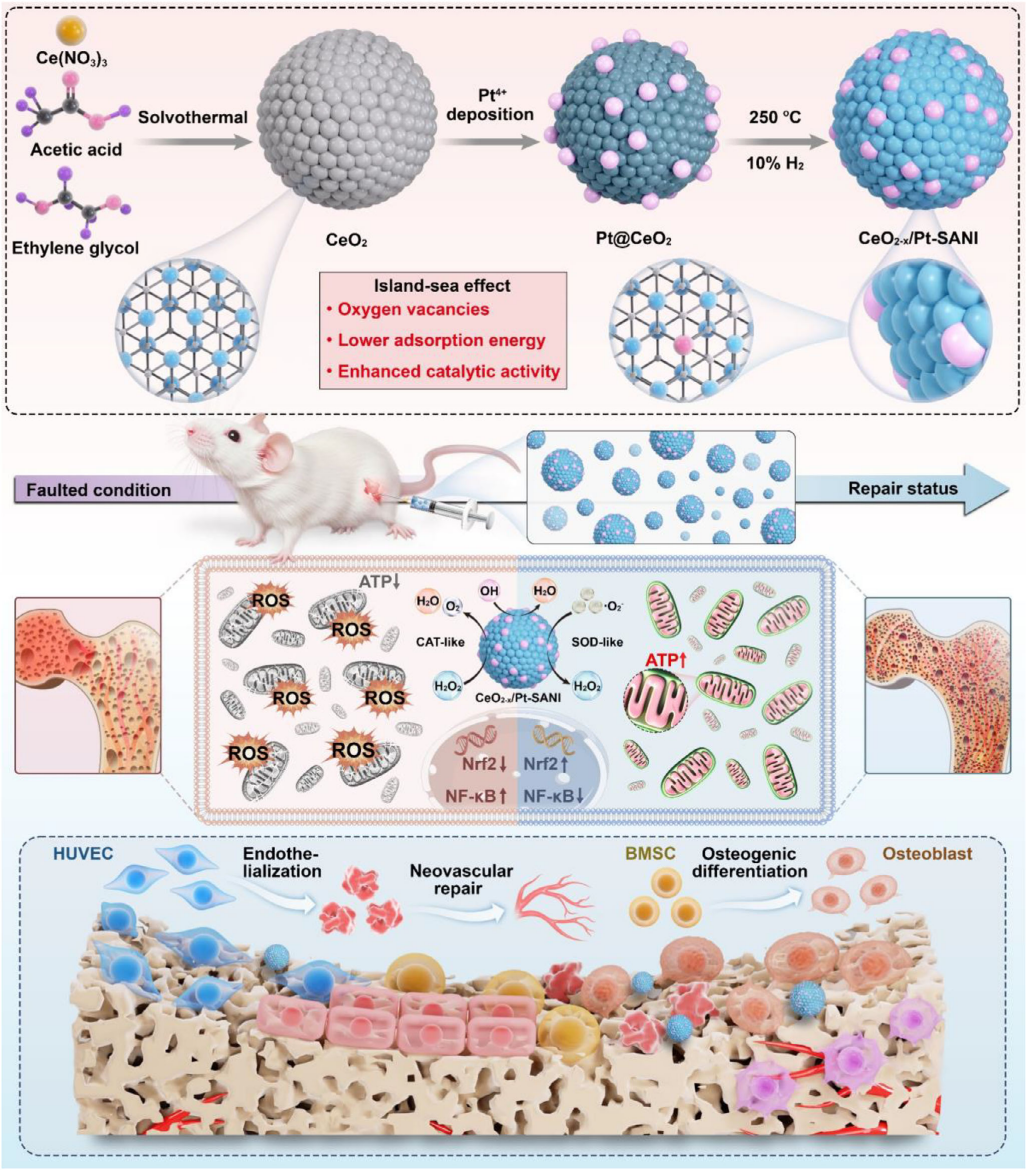
Schematic illustration of the synthesis and therapeutic mechanism of CeO_2‐x_/Pt SANI nanozyme in SONFH.

Here, noble metal platinum (Pt) single atoms are successfully embedded onto CeO_2_ nanoisland via low‐temperature in situ reduction, resulting in the fabrication of CeO_2‐x_/Pt SANI with plentiful oxygen vacancies and ultra‐high enzymatic activity (Figure 1). In this configuration, the surface CeO_2‐x_ (“islands”) serve as nano‐glue, effectively confining and stabilizing Pt single atoms, while the CeO_2_ support (“sea”) provides a robust platform that facilitates strong metal support electron transfer. While Pt‐based catalysts on CeO_2_ have been explored for oxidation catalysis, our work distinguishes itself in three key aspects: Design concept: Previous studies focused on thermal‐catalytic oxidation reactions (CO oxidation, etc.), while this work is dedicated to nanozyme catalysis for biomedical anti‐inflammation and cyto‐protection, a completely different application scenario; Structure and microenvironment: Our CeO_2‐x_/Pt SANI features abundant surface oxygen vacancies, lattice distortion, and strong metal‐support interaction tailored for ROS/RNS scavenging, rather than high‐temperature thermal catalysis; Mechanistic focus: We systematically elucidate multi‐enzyme‐mimetic activities, radical‐scavenging mechanisms, and in vivo therapeutic effects, which have not been reported in the cited Pt/CeO_2_ catalytic materials; Biological application: This work establishes the structure‐activity relationship between atomic Pt sites, oxygen vacancies, and cytoprotective effects under physiological conditions, which is absent in conventional heterogeneous catalysis studies.

Leveraging the unique charge‐transfer structures and the confinement effect of nanoislands, CeO_2‐x_/Pt SANI exhibits significantly enhanced •OH scavenging capacity, SOD‐like and CAT‐like activities compared to CeO_2_ nanozyme, attribute to the island‐sea synergistic effect that lowers energy barriers of reaction intermediates, as further confirmed by density functional theory (DFT) calculations. DFT calculations further demonstrate that Pt doping not only increases the number of oxygen vacancies but also bringing the d‐band center closer to the Fermi level (0 eV), which is conducive to the adsorption of ROS such as hydrogen peroxide (H_2_O_2_), •OH, and superoxide anions (· *O*_2_^−^), thereby promoting the reaction rate. Both in vitro and in vivo experiments conclusively validate that CeO_2‐x_/Pt SANI effectively reprograms the redox microenvironment in a SONFH model. Specifically, CeO_2‐x_/Pt SANI eliminates excessive ROS, restores mitochondrial homeostasis and reactivates the function of bone marrow stromal cells, collectively driving osteogenesis and angiogenesis and achieving impressive catalytic therapeutic outcomes. The innovative “island‐sea” structured SANs‐based therapeutic paradigm paves the way for novel treatments of osteonecrosis, forging a unique bridge between catalysis and regenerative medicine through an innovative “atomic catalyst‐bioregulation” approach.

## 2 Results and Discussion

### 2.1 Synthesis and Characterization of CeO_2‐x_/Pt SANI

**FIGURE 2:**
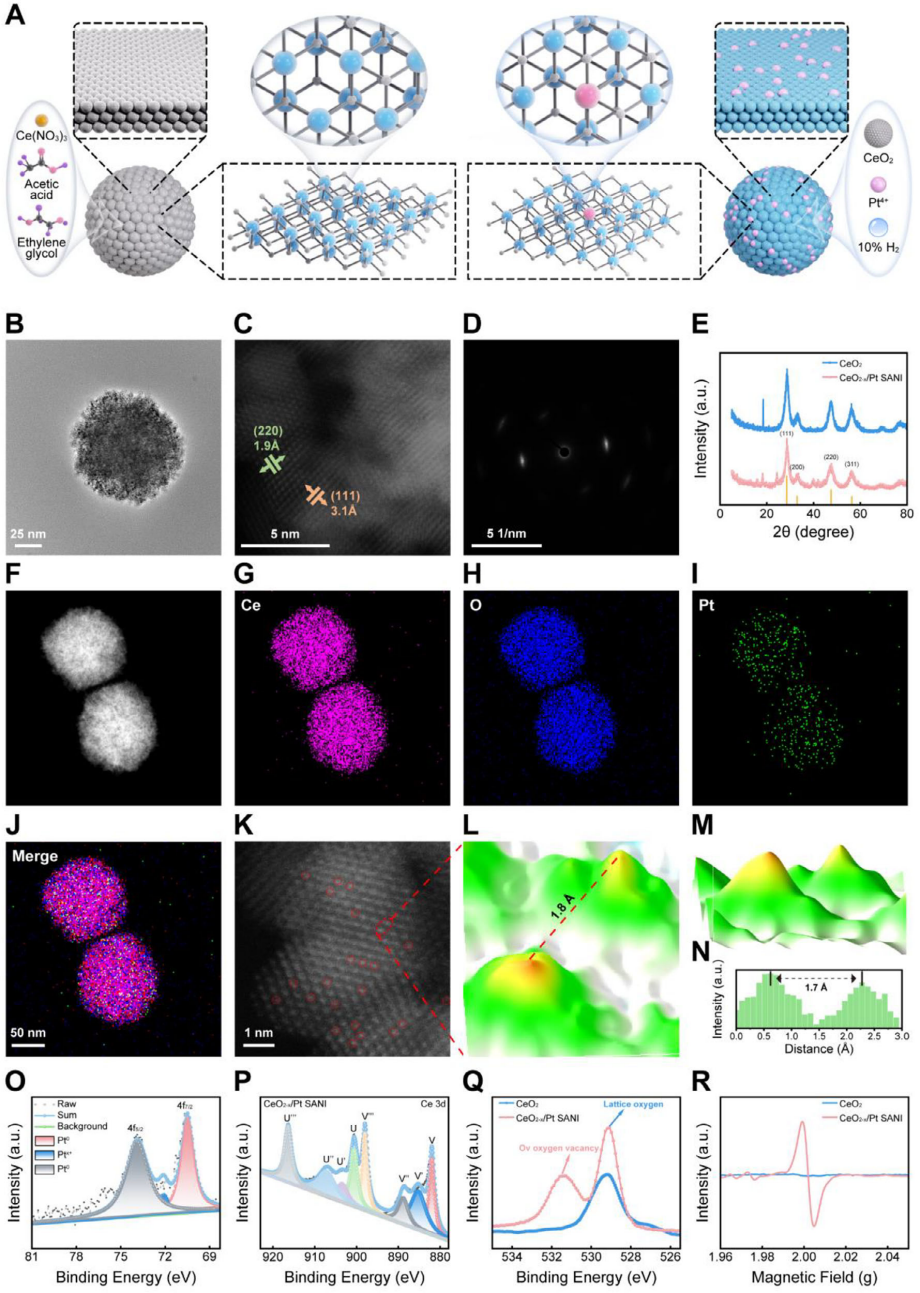
Characterizations of CeO_2‐x_/Pt SANI. (A) The structure diagram of CeO_2_ and CeO_2‐x_/Pt SANI (B) HR‐TEM image of CeO_2‐x_/Pt SANI. Scale bar = 20 nm. (C) AC HAADF‐STEM images of CeO_2‐x_/Pt SANI. Scale bar = 2 nm. (D) SAED image of CeO_2‐x_/Pt SANI. Scale bar = 5 1/ nm. (E) XRD patterns of CeO_2‐x_/Pt SANI and CeO_2_. (F–J) HAADF‐STEM images of CeO_2‐x_/Pt SANI. (K) AC HAADF‐STEM images of CeO_2‐x_/Pt SANI. The isolated Pt atoms are encircled with red circles. Scale bar = 1 nm. (L,M) 3D model of CeO_2‐x_/Pt SANI bright spots along the yellow dotted line and (N) the corresponding intensity profiles along the yellow circle. (O) High resolution XPS of Pt 4f and (P) Ce 3d in CeO_2‐x_/Pt SANI. (Q) XPS of O 1s in CeO_2‐x_/Pt SANI and CeO_2_. (R) ESR spectra of CeO_2‐x_/Pt SANI and CeO_2_.

The synthetic pathway for the fabrication of CeO_2‐x_/Pt SANI is illustrated in Figure 2A. Initially, the CeO_2_ support, which serves as the “sea”, is meticulously fabricated via a solvothermal, resulting in a nanostructure with an average diameter of approximately 100 nm (Figures S1 and S2). Subsequently, Pt atoms are absorbed and uniformly deposited onto CeO_2_ support. A crucial calcination step under hydrogen atmosphere is then employed to reduce the CeO_2_ and simultaneously anchor the Pt atoms, yielding the CeO_2‐x_/Pt SANI. As depicted in Figure 2B, transmission electron microscopy (TEM) image clearly demonstrates that that the synthesized CeO_2‐x_/Pt SANI exhibits a distinct spherical morphology characterized by a notably rough surface texture. High‐resolution TEM (HRTEM) image (Figure 2C and Figure S3) clearly delineates the lattice fringes corresponding to the (111) and (220) crystallographic planes of CeO_2_, confirming the crystalline nature of the support. Furthermore, the selected area electron diffraction (SAED) image of CeO_2‐x_/Pt SANI demonstrates the absence of any Pt clusters or small particles, indicating that the Pt atoms are highly dispersed throughout the structure (Figure 2D). X‐ray diffraction (XRD) patterns of both CeO_2_ and CeO_2‐x_/Pt SANI reveal that the main diffraction peaks are indexed to the cubic structure of CeO_2_ (Figure 2E). Notably, aside from the CeO_2_ peaks, no distinct diffraction peaks corresponding to aggregated Pt species are detected. This absence underscores that Pt exists as highly isolated single atoms rather than clusters or particles, and importantly, the introduction of Pt does not alter the underlying structure of CeO_2_. The XRD pattern of the as‑prepared Pt nanoparticles shows four characteristic diffraction peaks at 2θ = 39.76°, 46.24°, 67.45°, and 81.20°, which can be readily indexed to the (111), (200), (220), and (311) planes of the face‑centered cubic platinum. Energy dispersive spectroscopic (EDS) mapping analysis further corroborates the uniform distribution of the Pt component within the CeO_2‐x_/Pt SANI (Figure 2F–J). Additionally, aberration‐corrected high‐angle annular dark‐field scanning transmission electron microscopy (HAADF‐STEM) imaging visually highlights numerous isolated Pt single atoms (marked by red circles) embedded within the CeO_2‐x_ surface lattice, resembling “islands” of Pt atoms within the CeO_2_ “sea”. (Figure 2K). The inter‐atomic distance histogram shows that the distance between the two single‐atomic Pt is 1.7 Å, which is consistent with the expected distance between isolated Pt atoms, thereby confirming the exclusive presence of atomically dispersed Pt (Figure 2L–N). The Pt loading capacity in CeO_2‐x_/Pt SANI is determined to be approximately 1.4 wt.%, showcasing the high efficiency of Pt utilization. Raman analyses (Figure S4) show a characteristic peak centered at 460 cm^−1^ is ascribed to the F_2g_ vibrational mode of CeO_2_. When compared to pristine CeO_2_, the F_2g_ mode of CeO_2‐x_/Pt SANI is exhibits a notable softening, accompanied by the emergence of a broad feature centered around 600 cm^−1^. This new feature is attributed to a defect‐induced vibrational mode (denoted as the D band). The appearance of the D band is indicative of the formation of defect species within the Ce‐O coordination environment. This defect formation disrupts the symmetry of the Ce‐O vibrations, preventing their signals from being completely canceled out in all directions. Moreover, Raman peaks typically associated with the PtO_2_ phase, which would be expected at 504 and 545 cm^−1^, are conspicuously absent. This absence further underscores the absence of significant PtO_2_ phase crystalline phases within the CeO_2‐x_/Pt SANI. As shown in Figure S5, upon Pt doping, the surface charge of CeO_2_ nanoparticles shifts from negative to positive, providing direct evidence for the efficient incorporation of Pt species. These comprehensive characterizations offer invaluable and detailed insights into the structural integrity of CeO_2‐x_/Pt SANI, affirming its exceptional suitability and promising potential for a wide range of catalytic applications.

X‐ray photoelectron spectroscopy (XPS) was employed to probe the valence states of Pt, Ce and O in both CeO_2‐x_/Pt SANI and CeO_2_. The high‐resolution Pt 4f spectrum unveiled a distinct spin‐orbit doublet with binding energies at 70.5 eV (4f_7/2_) and 73.9 eV (4f_5/2_), indicative of the majority of Pt atoms residing in a zero‐valent and intermediate‐valent states (Figure 2O). Consistent with expectations, the Ce 3d XPS analysis reveals a significantly higher Ce^3+^/Ce^4+^ ratio in CeO_2‐x_/Pt SANI (Figure 2P) compared to that of CeO_2_ (Figure S6), confirming amplified electronic donation and catalytic performances. Furthermore, the increased presence of lower oxidation state Ce species is directly attributed to the deliberate introduction of oxygen vacancies. In contrast to the O 1s XPS spectrumin of CeO_2_, CeO_2‐x_/Pt SANI exhibits two distinct peaks at binding energy of 531.5 and 529.1 eV, corresponding to oxygen vacancies and lattice oxygen, respectively (Figure 2Q). To further substantiate the existence of these oxygen vacancies, electron spin resonance (ESR) spectroscopy was utilized. Figure 2R displays a clear ESR signal at a g value of 2.001, which is characteristic of electrons trapped in oxygen vacancies. These vacancies not only reduce the adsorption energy but also act as efficient conduits for electron storage and transport, thereby facilitating enhanced electron transfer and significantly boosting the catalytic efficiency. In addition, we monitored the hydrodynamic diameter of CeO_2‐x_/Pt SANI when incubated in both DMEM and FBS over a 96 h period. The results demonstrate that the particle size remained remarkably stable and consistent (approximately between 120 nm and 130 nm) throughout the entire incubation (Figure S7). This indicates excellent colloidal stability of our CeO_2‐x_/Pt SANI in complex physiological environments, preventing aggregation or significant degradation.

**FIGURE 3:**
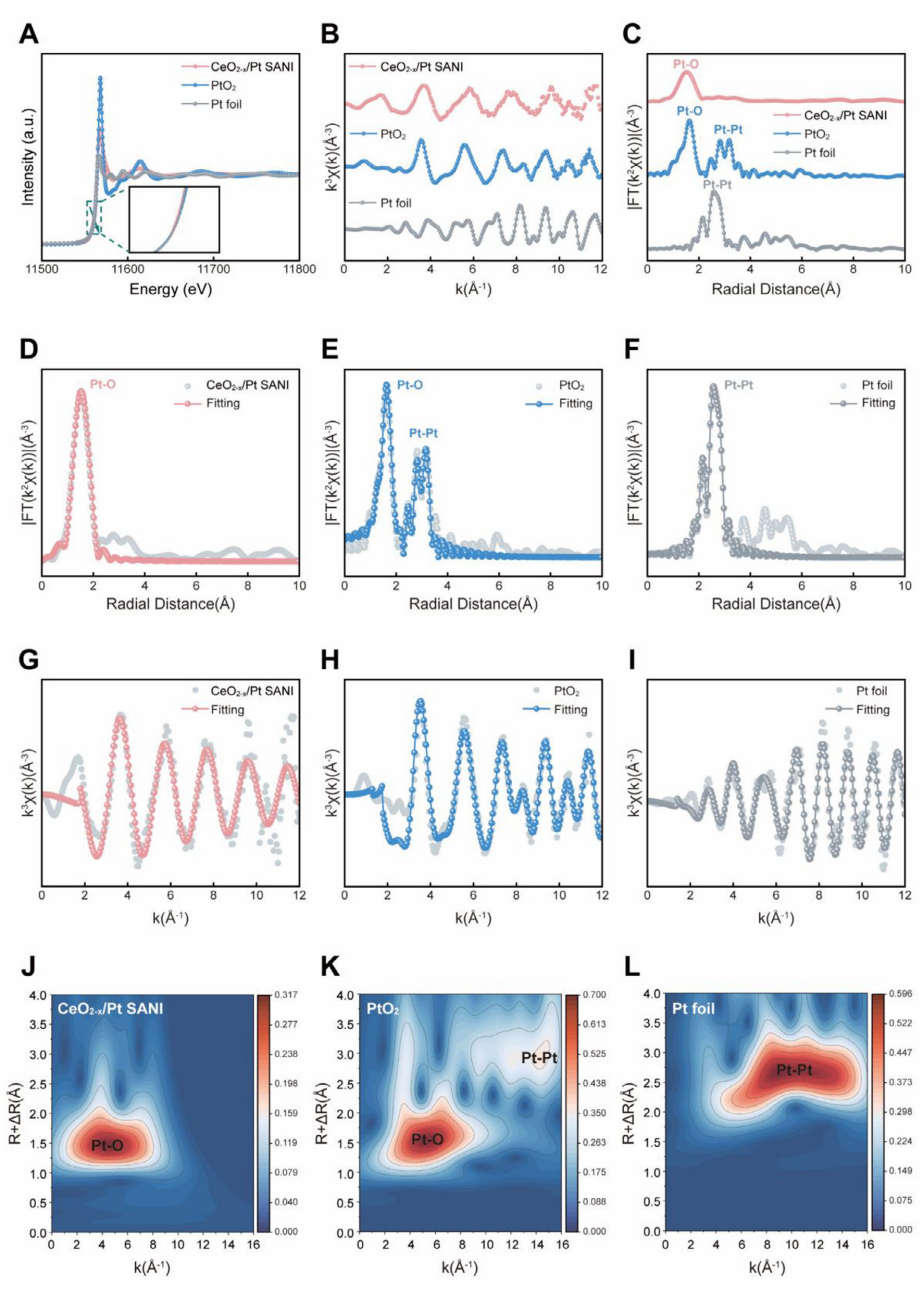
Characterizations of CeO_2‐x_/Pt SANI. (A) XANES spectra and matching (B) Fourier transform EXAFS spectra of CeO_2‐x_/Pt SANI. (C) EXAFS curves of CeO_2‐x_/Pt SANI at the k space. (D–F) EXAFS fitting curve of CeO_2‐x_/Pt SANI, PtO_2_ and Pt foil at the R space. (G–I) EXAFS fitting curve of CeO_2‐x_/Pt SANI, PtO_2_ and Pt foil at the k space. (J–L) WT of CeO_2‐x_/Pt SANI, PtO_2_ and Pt foil.

To elucidate the electronic structure and coordination environment of Pt atoms within CeO_2‐x_/Pt SANI, synchrotron radiation‐based X‐ray absorption near‐edge structure (XANES) spectroscopy and extended X‐ray absorption fine structure (EXAFS) are conducted. Pt K‐edge XANES spectra positioned the absorption threshold of CeO_2‐x_/Pt SANI between those of PtO_2_ and Pt foil (Figure 3A), thereby corroborating the Pt^δ+^ (0<δ<4) assignment obtained from XPS. This positioning confirms the intermediate oxidation state of Pt within CeO_2‐x_/Pt SANI. Fourier‐transformed (FT) EXAFS in R‐space reveals prominent peaks at 1.50 Å, corresponding to Pt‐O bonds (Figure 3B–F). These peaks provide definitive evidence of the presence of atomically dispersed Pt single atoms within CeO_2‐x_/Pt SANI. Quantitative EXAFS fitting analysis discloses that each Pt species center is coordinated by four O atoms (Figure 3G–I and Table S1), indicating a tetrahedral coordination environment. Moreover, EXAFS wavelet transforms (WT) analysis of CeO_2‐x_/Pt SANI provided additional insights into the unique coordination structure of the Pt‐O bond, with a characteristic peak at 4.77 Å^−1^, which significantly distinct from those observed in bulk PtO_2_ and Pt foil (Figure 3J–L). Collectively, these comprehensive findings definitely indicate that single‐atomic Pt are successfully incorporated into the oxygen vacancies of CeO_2‐x_/Pt SANI, forming Pt‐O coordination structures.

### 2.2 Catalytic Performance Evaluation of CeO_2‐x_/Pt SANI

**FIGURE 4:**
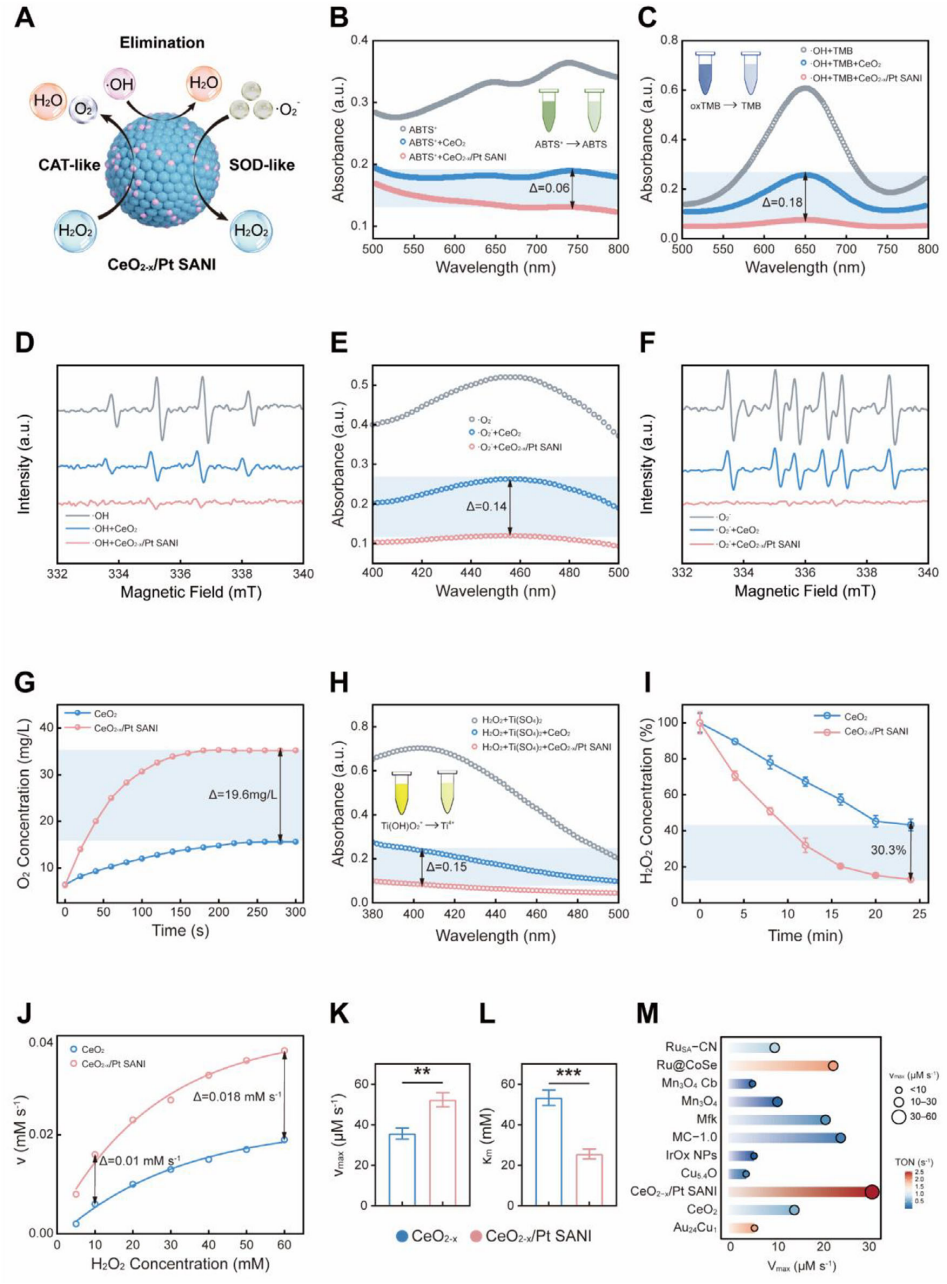
Evaluation of multi‐enzymatic ROS‐scavenging activities. (A) Schematic representation of catalysis with multienzyme‐like activities of •OH elimination, SOD‐like and CAT‐like reactions. (B) Ultraviolet‐visible (UV–vis) spectra of ABTS and (C) TMB following incubation with Fe^3+^ and H_2_O_2_ in the presence of CeO_2‐x_/Pt SANI and CeO_2_. (D) ESR curves of •OH captured using a DMPO in the presence of CeO_2‐x_/Pt SANI and CeO_2_. (E) UV–vis spectra of WST‐8 in the presence of CeO_2‐x_/Pt SANI and CeO_2_. (F) ESR curves of •O_2_^−^ captured using a DMPO in the presence of CeO_2‐x_/Pt SANI and CeO_2_. (G) Oxygen generation rates of H_2_O_2_ decomposed by CeO_2‐x_/Pt SANI and CeO_2_, as measured using dissolved oxygen analyzer. (H) UV–vis spectra of Ti(SO_4_)_2_ in the presence of CeO_2‐x_/Pt SANI and CeO_2_. (I) Time‐dependent CAT‐like performances via TiSO_4_‐based method with the presence of CeO_2‐x_/Pt SANI or CeO_2_ plus H_2_O_2_ (n = 3). (J) Michaelis‐Menten kinetic analysis of CeO_2‐x_/Pt SANI or CeO_2_ with H_2_O_2_ as substrate (*n* = 3). (K) V_max_ value of CeO_2‐x_/Pt SANI and CeO_2_ (*n* = 3). (L) K_m_ value of CeO_2‐x_/Pt SANI and CeO_2_ (*n* = 3). (M) Comparison and analysis of the TON and V_max_ values with previously reported nanozyme. Data are presented as mean ± SEM. Two‐tailed Student's t‐test was used for comparisons. **p < 0.01, ***p < 0.001.

A thorough enzymatic evaluation was conducted on both CeO_2_ and CeO_2‐x_/Pt SANI, with a focusing on SOD‐like, CAT‐like activities as well as their efficacy in scavenging •OH (Figure 4A). The capacity to scavenge radical nitrogen species (RNS) was assessed using 2,2’‐azinobis (3‐ethylbenzothiazoline‐6‐sulfonic acid) diammonium salt (ABTS). As depicted in Figure 4B and Figure S8, CeO_2‐x_/Pt SANI demonstrate a significantly higher RNS scavenging capacity compared to CeO_2_, in a manner that was dose‐dependent. We have performed additional experiments to detect peroxynitrite (ONOO•) scavenging activity using a fluorescent probe. The results demonstrate that CeO_2‐x_/Pt SANI exhibits excellent peroxynitrite‐scavenging ability, further verifying its efficient RNS removal capacity (Figure S9). This suggests that the presence of oxygen vacancies and the incorporation of platinum in CeO_2‐x_/Pt SANI markedly enhance its catalytic performance. The •OH scavenging activity was measured using the chromogenic probe 3,3’,5,5’‐tetramethylbenzidine (TMB). The rate of absorbance decrease at 652 nm was significantly faster for CeO_2‐x_/Pt SANI than for CeO_2_, highlighting its superior catalytic efficacy, which can be attributed to its unique nanoisland configuration (Figure 4C and Figure S10). This finding was further supported by ESR spectroscopy, using 5,5‐dimethyl‐1‐pyrrole oxide (DMPO) as a spin trap. The quartet signal corresponding to the DMPO/•OH intermediate (1:2:2:1) was almost entirely quenched by CeO_2‐x_/Pt SANI, whereas a pronounced signal remained with CeO_2_, indicating the amplified •OH scavenging capacity of CeO_2‐x_/Pt SANI (Figure 4D). The SOD‐like activity was measured using a WST‐8 assay. CeO_2‐x_/Pt SANI eliminated approximately 77% of •O_2_^−^ within 10 min, significantly outperforming CeO_2_ (Figure 4E and Figure S11). Notably, even at an extremely low concentration of 10 µg mL^−1^, CeO_2‐x_/Pt SANI still removed about 30% of •O_2_^−^ (Figure S12). This enhancement was further validated by ESR spin‐trapping with DMPO: the sextet signal of the DMPO/•O_2_^−^ adduct was markedly reduced by CeO_2‐x_/Pt SANI in comparison to CeO_2‐x_ (Figure 4F).

We subsequently investigated the CeO_2‐x_/Pt SANI in the decomposition of H_2_O_2_. Utilizing a dissolved‐oxygen analyzer, we meticulously monitored in situ generation of O_2_. Upon the addition of H_2_O_2_, CeO_2‐x_/Pt SANI remarkably elevates O_2_ concentration from 7.2 to 31.3 mg mL^−1^ within a mere 40 s, significantly outperforming CeO_2_ (Figure 4G). The reaction kinetics are elucidated through a spectrophotometrically analysis utilizing a Ti(SO_4_)_2_ kit. The absorbance at 410 nm of the yellow Ti(OH)O_2_^+^ complex diminishes at a significantly faster rate in the presence of CeO_2‐x_/Pt SANI compared to that of CeO_2_, confirming its higher CAT‐mimicking activity (Figure 4H and Figure S13). After a 20 min reaction period, CeO_2‐x_/Pt SANI maintains at least 95% of its H_2_O_2_‐decomposition capacity, and it shows virtually no activity loss across four consecutive reaction cycles, highlighting profound reusability and operational stability (Figure 4I). Michaelis‐Menten analysis reveal that CeO_2‐x_/Pt SANI shows a maximal velocity (Vmax) of 52.3 µM s^−1^ and a Michaelis constant (Km) of 25.6 mM (Figure 4J–L). These values significantly outperform those of the benchmark CeO_2_, which has a Vmax of 35.67 µM s^−1^ and Km of 53.4 mM. As a result, CeO_2‐x_/Pt SANI achieves the comparatively high turnover number (TON) currently reported for CAT‐like activity (Figure 4M and Table S2). We have performed recycling experiments to evaluate the stability and reusability of the nanozyme. The results show that the catalyst retains high CAT‐like activities after repeated cycles, indicating good structural stability under oxidative conditions (Figure S14). We conducted a O_2_ concentration comparing CeO_2‐x_/Pt SANI with other reported nanozymes (MnO_2_, Fe_2_O_3_, V_2_O_5_). As shown in Figure S15, CeO_2‐x_/Pt SANI exhibited significantly higher CAT‐like activity, indicating its superior catalytic efficiency. As depicted in Figures S16–S18, CeO_2‐x_/Pt SANI displayed superior catalytic activity in PBS, whereas its activity was substantially suppressed in DMEM and whole blood due to the adsorption of serum proteins that block the catalytic active sites. Upon dispersion in serum‐containing media, nanoparticles readily adsorb serum proteins to form a protein corona, which can alter hydrodynamic size, surface charge, colloidal stability, cellular uptake, and in vivo biodistribution. For our system, we acknowledge that serum proteins may slightly increase particle size and reduce surface potential. However, the core structural integrity and catalytic activity of our nanoparticles remain largely preserved under physiological conditions. Meanwhile, the optimal catalytic performance was achieved at physiological pH 7.4, which was remarkably higher than those at acidic pH 5.0 and alkaline pH 9.0. Collectively, these comprehensive and compelling findings firmly establish CeO_2‐x_/Pt SANI as a highly robust and broad‐spectrum catalytic activity, endowed with exceptional activity. This positions it as an exceptionally promising candidate for the treatment of SONFH, offering significant potential for therapeutic applications.

**FIGURE 5:**
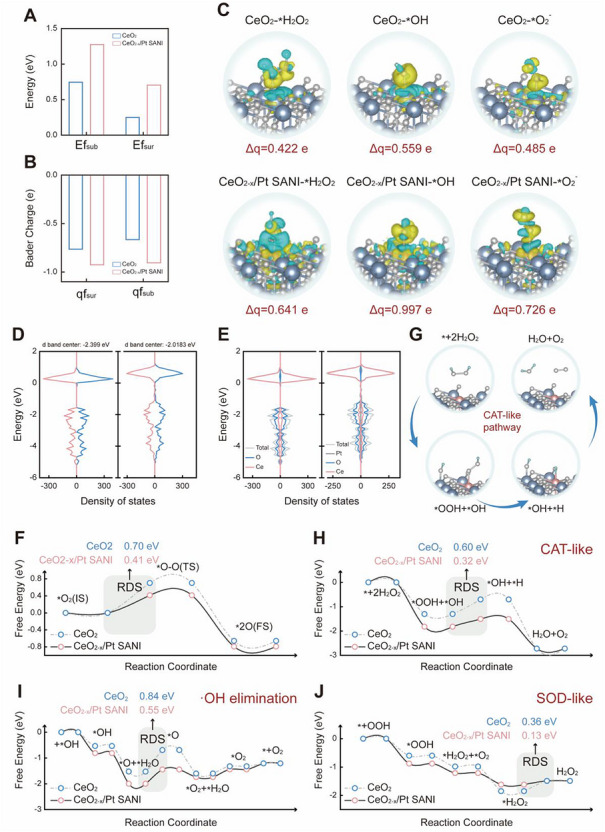
DFT calculation and catalytic mechanism of CeO_2_ and CeO_2‐x_/Pt SANI. (A) The Bader charges of oxygen formed on the surface and subsurface of CeO_2_ and CeO_2‐x_/Pt SANI. (B) The formation energies of oxygen vacancies on the surface and subsurface of CeO_2_ and CeO_2‐x_/Pt SANI. (C) The CDD and Bader charge of CeO_2_ (up) and CeO_2‐x_/Pt SANI (down) structures upon adsorption of *H_2_O_2_, *•OH, and *•O_2_^−^ with an isosurface value set at 0.003 e/Å^3^, yellow represents the accumulation area of electrons, while blue represents the dissipation area of electrons. (D) DOS analysis of Ce, C, O, and Pt atoms in CeO_2_ and CeO_2‐x_/Pt SANI. (E) DOS analysis of d band in CeO_2_ and CeO_2‐x_/Pt SANI. (F) The energy barriers for O_2_ adsorption and dissociation to form 2O in CeO_2_ and CeO_2‐x_/Pt SANI structures. (G) Diagram for the CAT‐like reaction mechanism of CeO_2‐x_/Pt SANI. (H) Gibbs free‐energy diagrams for the decomposition of •OH into H_2_O, (I) the conversion of •O_2_^−^ into H_2_O_2_, and (J) the conversion of H_2_O_2_ into O_2_ on CeO_2_ and CeO_2‐x_/Pt SANI.

To unravel the intrinsic mechanisms underpinning the enhanced catalytic activity induced by “island‐sea” effect, we conduct DFT calculations. The geometrically optimized structures of CeO_2‐x_/Pt SANI and CeO_2_ are depicted in Figure S19. CeO_2‐x_/Pt SANI features an oxygen vacancies‐proximal Pt‐O_4_ single‐atom configuration. This unique arrangement induces structural polarization, significantly enhancing catalytic performances (Figure 5A). Then, we analyze the Bader charges for O atoms on the surface and sub‐surface. The surface O atoms exhibit a lower charge compared to those on the sub‐surface, indicating an electron‐deficient state that facilitates their involvement in reactions. This phenomenon is more pronounced in the CeO_2‐x_/Pt SANI structure, confirming that Pt doping significantly induced oxygen vacancy, thereby modulating the electron structure (Figure 5B,C). Charge density difference reveals that the CeO_2‐x_/Pt SANI shows higher charge density for H_2_O_2_, •OH and O_2_ adsorption, along with enhanced electron transfer, signifying stronger adsorption capabilities. Thus, Pt doping strengthens the adsorption of H_2_O_2_, •OH, and O_2_, thereby boosting the reaction. Moreover, we analyze density of states (DOS) d‐band center shifts for CeO_2_ and CeO_2‐x_/Pt SANI structures. Upon Pt doping, the d‐band center moves closer to the Fermi level (0 eV) due to increased electron density. This enhances the adsorption capacity of CeO_2‐x_/Pt SANI, raises the surface electron count, and boosts overall reactivity (Figure 5D). Energy barrier calculations for O_2_ adsorption and desorption (leading to 2O formation) demonstrated that Pt atomic cluster introduction promotes O_2_ desorption, reducing the energy barrier and enhancing catalytic activity of CeO_2‐x_/Pt SANI (Figure 5E). Additionally, the rate‐determining step (RDS) for O_2_ → 2O conversion is identified as the cleavage of the O‐O bond on the catalyst surface: CeO_2‐x_/Pt SANI exhibited a significantly lower energy barrier for this step (0.41 eV) compared to CeO_2_ (0.70 eV) (Figure 5F and Figure S20).

The catalytic pathways mediated by CeO_2_ and CeO_2‐x_/Pt SANI are depicted herein (Figure 5G). For CAT‐like activity, the RDS‐reduction of the adsorbed *OOH+ *OH to form adsorbed *OH+ *H at the CeO_2‐x_/Pt SANI active site‐exhibits an energy decreases of 0.32 eV, which is greatly lower than the 0.6 eV barrier on the CeO_2_ catalytic site (Figure 5H and Figure S21). This proves that CeO_2‐x_/Pt SANI more effectively promotes the reduction of *OOH+ *OH and subsequent O_2_ desorption. Given •OH was identified as a ROS during catalysis, the •OH‐scavenging catalytic process catalytic process can be divided into six sequential steps: 1) adsorption of •OH on the nanozyme surface (+*OH); 2) reduction of the surface‐adsorbed 2*OH to form adsorbed *O and H_2_O at the catalytic sites (*O+ *H_2_O). 3) (RDS) desorption of H_2_O from the catalytic sites (*O). 4) reduction of adsorbed *O to generate adsorbed *O_2_+ H_2_O at the catalytic sites (*O_2_+ H_2_O). 5) desorption of H_2_O from the catalytic sites (+ *O_2_). 6) desorption of *O_2_ from the active sites, thereby regenerating the catalyst. The RDS for H_2_O desorption at the Pt‐O_4_ active site of CeO_2‐x_/Pt SANI has an energy decrease of 0.55 eV, remarkably lower than 0.84 eV barrier of CeO_2_ (Figure 5I and Figure S22), demonstrating that CeO_2‐x_/Pt SANI facilitated H_2_O release more readily. Similarly, for the conversion of •O_2_^−^ to H_2_O_2_, the RDS is identified as H_2_O_2_ desorption from catalyst surface. CeO_2‐x_/Pt SANI shows a lower energy barrier for this step (0.13 eV) compared to CeO_2_ (0.36 eV) (Figure 5J and Figure S23), suggesting it is more favorable for H_2_O_2_ desorption and subsequent catalyst reactivation. Collectively, these DFT calculation results indicate that CeO_2‐x_/Pt SANI structure, engineered via the synergistic effect of the “island‐sea” architecture and Pt doping‐induced oxygen vacancies, effectively strengthens catalytic activity.

### 2.3 CeO_2‐x_/Pt SANI Alleviates Oxidative Damage and Rejuvenates Osteogenic and Angiogenic Capacities in SONFH

**FIGURE 6:**
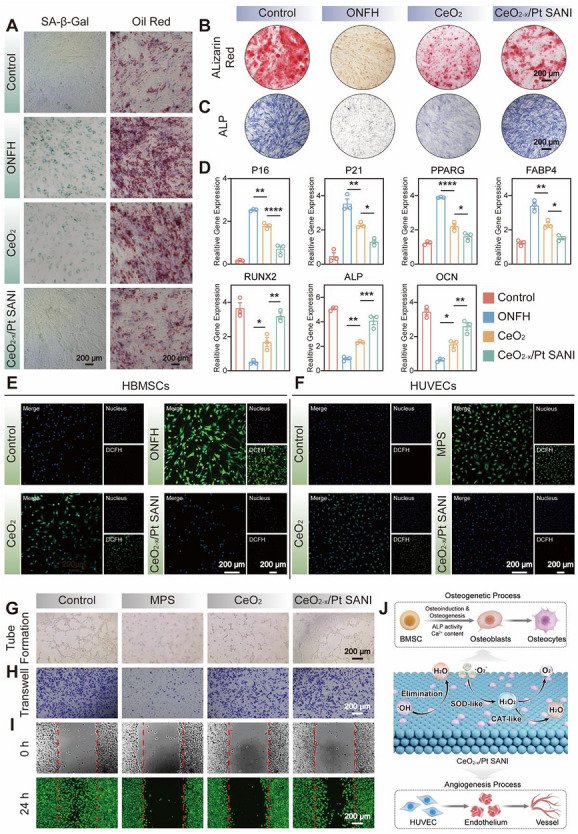
CeO_2‐x_/Pt SANI rejuvenates osteogenic and angiogenic function in SONFH. (A) SA‐β‐Gal and Oil Red O staining of HBMSCs subjected to SONFH and treatments. (B‐C) Alizarin Red and ALP staining assess osteogenic differentiation. (D) qRT‐PCR analysis of senescence‐associated (P16 and P21), adipogenic (PPARG and FABP4) and osteogenesis‐related genes (RUNX2, ALP and OCN). (*n* = 3). (E,F) Immunofluorescence evaluation of HBMSCs and HUVECs under various treatments. (G) Tube formation assay of endothelial cells. (H) Endothelial cell migration assay via Transwell chambers. (I) Wound healing assay demonstrating endothelial repair capacity at 0 and 24 h. (J) Mechanistic schematic illustrating CeO_2‐x_/Pt SANI mediated redox reprogramming in SONFH. Data are presented as mean ± SD. One‐way ANOVA with Tukey's post hoc test was used for multiple comparisons. *p < 0.05, **p < 0.01, ***p < 0.001, ****p < 0.0001.

Glucocorticoid‐induced oxidative stress drives cellular senescence and inhibits osteogenesis, contributing to SONFH pathogenesis. In our in vitro SONFH model, CeO_2‐x_/Pt SANI effectively alleviated oxidative damage by reducing cellular senescence and pathological adipogenesis while restoring osteogenic potential. SONFH conditions markedly increased senescence‐associated β‐galactosidase (SA‐β‐Gal) activity and lipid accumulation in HBMSCs, indicating elevated senescence and adipogenic differentiation. Treatment with CeO_2‐x_/Pt SANI significantly attenuated these changes, as evidenced by decreased SA‐β‐Gal staining and fewer Oil Red O‐positive lipid droplets (Figure 6A).

Osteogenic differentiation assays further demonstrated that CeO_2‐x_/Pt SANI restored the mineralization capacity of HBMSCs under oxidative stress. Alizarin Red S staining revealed abundant mineralized nodules in CeO_2‐x_/Pt SANI‐treated cells, and alkaline phosphatase (ALP) staining showed higher ALP activity compared to the untreated SONFH group (Figure 6B,C). Quantitative analyses confirmed these observations as ALP enzymatic activity, which was significantly suppressed by glucocorticoid exposure, was markedly increased by CeO_2‐x_/Pt SANI, exceeding the recovery afforded by CeO_2_ alone (Figure S24). Likewise, Alizarin Red absorbance demonstrated a strong rescue of mineral deposition in the CeO_2‐x_/Pt SANI group (Figure S25). Together, these data indicate that CeO_2‐x_/Pt SANI reverses the impairment of osteoblastic differentiation caused by glucocorticoids, with superior efficacy compared to CeO_2_.

Molecular analyses corroborated the phenotypic rescue conferred by CeO_2‐x_/Pt SANI. Quantitative reverse transcription polymerase chain reaction (qRT‐PCR) showed that SONFH conditions induced strong upregulation of cell‐cycle inhibitors P16 and P21 as well as adipogenic transcription factors PPARG and FABP4, relative to healthy controls (Figure 6D). CeO_2‐x_/Pt SANI treatment significantly downregulated these genes toward baseline levels, indicating reduced senescence and suppression of aberrant adipogenesis. Concurrently, CeO_2‐x_/Pt SANI elevated the expression of osteogenic markers including RUNX2, ALP and OCN, compared to untreated SONFH cells (Figure 6D). This coordinated shift in gene expression demonstrates that CeO_2‐x_/Pt SANI reprogrammed the cellular state from a degenerative, senescent phenotype toward an osteogenic, regenerative phenotype. Notably, this dual action, mitigating senescence and adipogenesis while promoting osteogenesis, addresses a key pathology of SONFH, wherein excessive marrow fat accumulation occurs at the expense of bone formation.

Angiogenesis is indispensable for bone repair, and CeO_2‐x_/Pt SANI also rescued endothelial function that was impaired under SONFH‐mimicking oxidative stress. Immunofluorescence assays showed improved cell morphology and viability in both HBMSCs and human umbilical vein endothelial cells (HUVECs) co‐cultured under glucocorticoid stress when CeO_2‐x_/Pt SANI was present (Figure 6E,F). In contrast, untreated cells exhibited shrunken, stressed morphology and reduced viability. Functional angiogenic assays further confirmed the pro‐regenerative effect of CeO_2‐x_/Pt SANI on vascular cells. Endothelial tube formation on Matrigel was severely impaired by SONFH conditions, yielding sparse and rudimentary networks. However, CeO_2‐x_/Pt SANI treatment markedly enhanced network complexity and length (Figure 6G). Quantitative analysis supported this improvement, with the CeO_2‐x_/Pt SANI group showing significantly more network nodes and junctions than the untreated SONFH and CeO_2_ groups (Figure S26A,B). Similarly, Transwell assays demonstrated that CeO_2‐x_/Pt SANI significantly promoted endothelial cell migration and invasion compared to untreated SONFH cells, restoring the migration rate toward near‐normal levels (Figure 6H and Figure S27). In wound‐healing scratch assays, CeO_2‐x_/Pt SANI accelerated endothelial monolayer repair over 24 h relative to the untreated condition (Figure 6I). At the molecular level, CeO_2‐x_/Pt SANI also rescued the expression of angiogenic factors. SONFH stress normally suppresses such factors, but ANG1 and VEGF were upregulated in CeO_2‐x_/Pt SANI‐treated endothelial cells, approaching levels seen in healthy controls (Figure S28A,B). Taken together, these findings demonstrate that CeO_2‐x_/Pt SANI substantially improves the angiogenic capacity of cells under oxidative stress.

The pronounced pro‐osteogenic and pro‐angiogenic effects of CeO_2‐x_/Pt SANI can be attributed to its potent and sustained catalytic activity. Excess reactive oxygen species disrupt redox‐sensitive signaling pathways, leading to cellular senescence, osteogenic dysfunction, and endothelial impairment. By continuously neutralizing ROS, CeO_2‐x_/Pt SANI preserves the signaling balance required for both osteoblast differentiation and angiogenic responses. This mechanism is illustrated in our schematic model where the “island‐sea” nanoarchitecture of CeO_2‐x_/Pt SANI enhances redox activity by facilitating electron transfer and ROS decomposition (Figure 6J).

### 2.4 CeO_2‐x_/Pt SANI Promotes Bone Regeneration and Functional Recovery In Vivo

**FIGURE 7:**
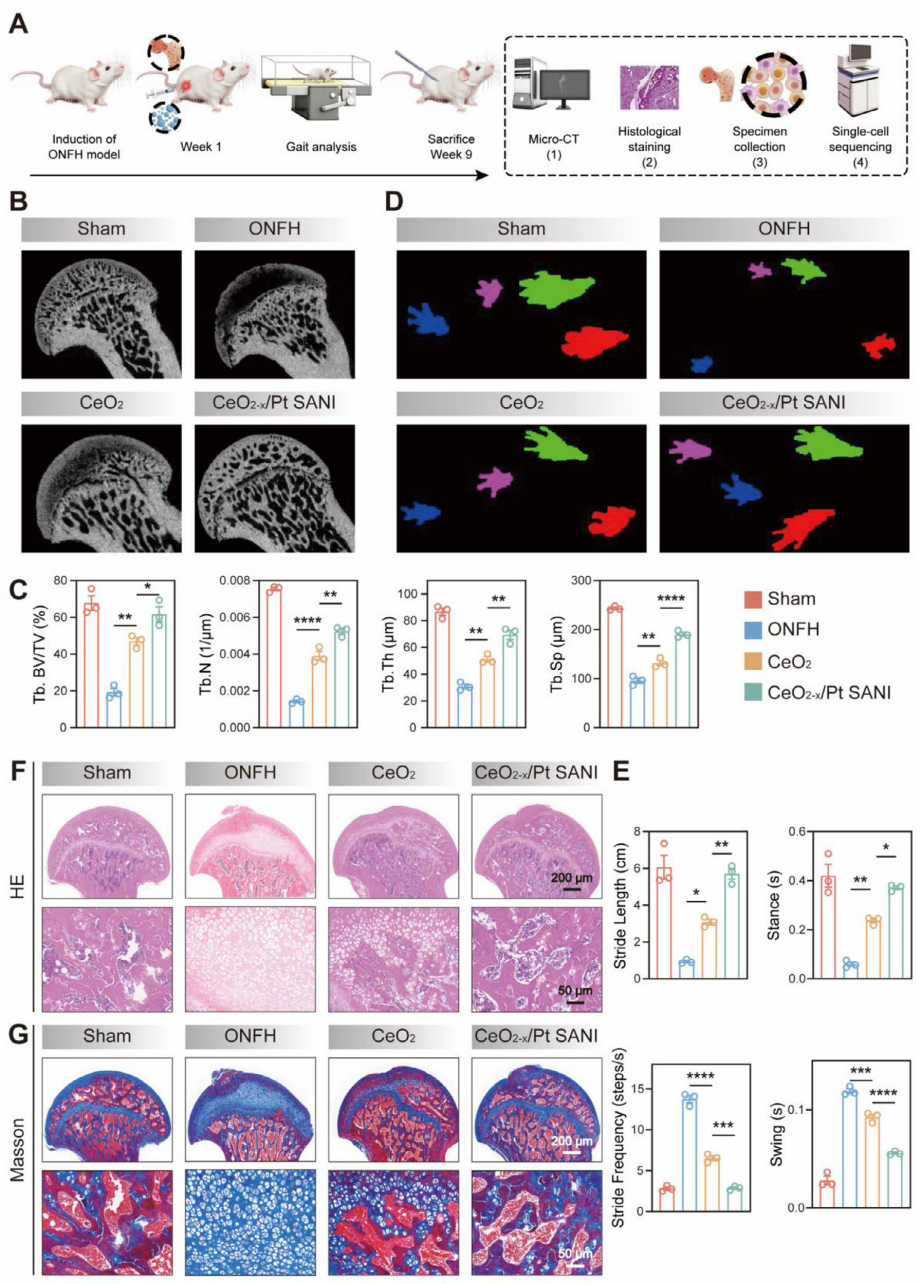
CeO_2‐x_/Pt SANI enhances bone regeneration and functional recovery in a SONFH rat model. (A) Schematic diagram of the experimental timeline and treatment design. (B) Representative micro‐CT images showing trabecular bone architecture in the femoral head across Sham, ONFH, CeO_2_ and CeO_2‐x_/Pt SANI groups. (C) Quantitative micro‐CT analysis of BV/TV, Tb.N, Tb.Th, and Tb.Sp. (*n* = 3). (D,E) Gait analysis including stride length, stance time, stride frequency and swing time to assess motor function recovery. (*n* = 3). (F) H&E staining showing histological morphology and bone marrow integrity. (G) Masson's trichrome staining illustrating collagen deposition and bone matrix regeneration. Data are presented as mean ± SD. One‐way ANOVA with Tukey's post hoc test was used for multiple comparisons. *p < 0.05, **p < 0.01, ***p < 0.001, ****p < 0.0001.

We next evaluated the therapeutic efficacy of CeO_2‐x_/Pt SANI in an in vivo rat model of SONFH (Figure 7A). CeO_2‐x_/Pt SANI treatment significantly preserved trabecular bone mass and microarchitecture in osteonecrotic femoral heads, leading to markedly improved bone regeneration compared to untreated or CeO_2_‐treated groups. Micro‐CT analysis revealed that glucocorticoid‐induced ONFH caused severe trabecular bone loss and structural deterioration in the femoral head (Figure 7B). Specifically, the SONFH group exhibited a drastic reduction in trabecular bone volume fraction (BV/TV), trabecular number (Tb.N), and trabecular thickness (Tb.Th), along with a corresponding increase in trabecular separation (Tb.Sp), relative to healthy sham controls (Figure 7C). Importantly, H&E staining of major organs and blood biochemistry analysis revealed no signs of inflammation, necrosis, hemorrhage or other pathological alterations were observed in any organ across all treatment groups (Figures S29 and S30). These results demonstrate that CeO_2‐x_/Pt SANI preserves bone structure under osteonecrotic conditions, consistent with its role as a protective antioxidant.

Improvements in bone structure translated into better limb function, as evidenced by gait analysis. CeO_2‐x_/Pt SANI‐treated rats showed significantly enhanced locomotor function compared to untreated SONFH rats, indicating functional recovery accompanying the structural bone healing (Figure 7D). Detailed gait metrics confirmed that SONFH induced pronounced gait impairments, as stride length and stance times were reduced, while stride frequency and swing times were abnormally prolonged, reflecting pain and instability in weight‐bearing. Remarkably, rats receiving CeO_2‐x_/Pt SANI exhibited near‐normal gait patterns. Their stride lengths were longer and stride frequencies lower than those of untreated SONFH animals, and their stance and swing times approached normal ranges (Figure 7E). These functional improvements correlated with the restored bone integrity in CeO_2‐x_/Pt SANI‐treated femoral heads. By contrast, rats treated with CeO_2_ nanoparticles alone showed only modest gait improvements, again demonstrating the advantage conferred by the Pt single‐atom nanozyme design.

Histological analysis provided further validation of CeO_2‐x_/Pt SANI's bone‐regenerative effects. Treated femoral heads displayed robust new bone formation and reduced marrow adiposity, reflecting healthier bone remodeling compared to untreated lesions. Hematoxylin and eosin (H&E) staining of SONFH samples revealed necrotic bone trabeculae, fatty marrow infiltration and few active osteoblasts. In sharp contrast, CeO_2‐x_/Pt SANI‐treated samples showed restored trabecular architecture with viable osteocytes and far fewer adipocytes in the marrow space, indicating a reversal of the fat‐laden, osteoblast‐deficient pathology characteristic of SONFH (Figure 7F). Likewise, Masson's trichrome staining demonstrated extensive collagen‐rich new bone matrix in the CeO_2‐x_/Pt SANI group, whereas the SONFH group exhibited only scant collagen and large fatty or necrotic areas (Figure 7G). These histological findings align with the micro‐CT data, confirming that CeO_2‐x_/Pt SANI stimulates new bone formation while inhibiting the aberrant adipogenesis and bone loss induced by glucocorticoids. Collectively, the in vivo results show that CeO_2‐x_/Pt SANI not only preserves bone structure but also actively promotes true tissue regeneration, culminating in restored mechanical function of the femoral head.

### 2.5 CeO_2‐x_/Pt SANI Accelerates Osteogenesis and Angiogenesis by Promoting Mineralization and Revascularization

**FIGURE 8:**
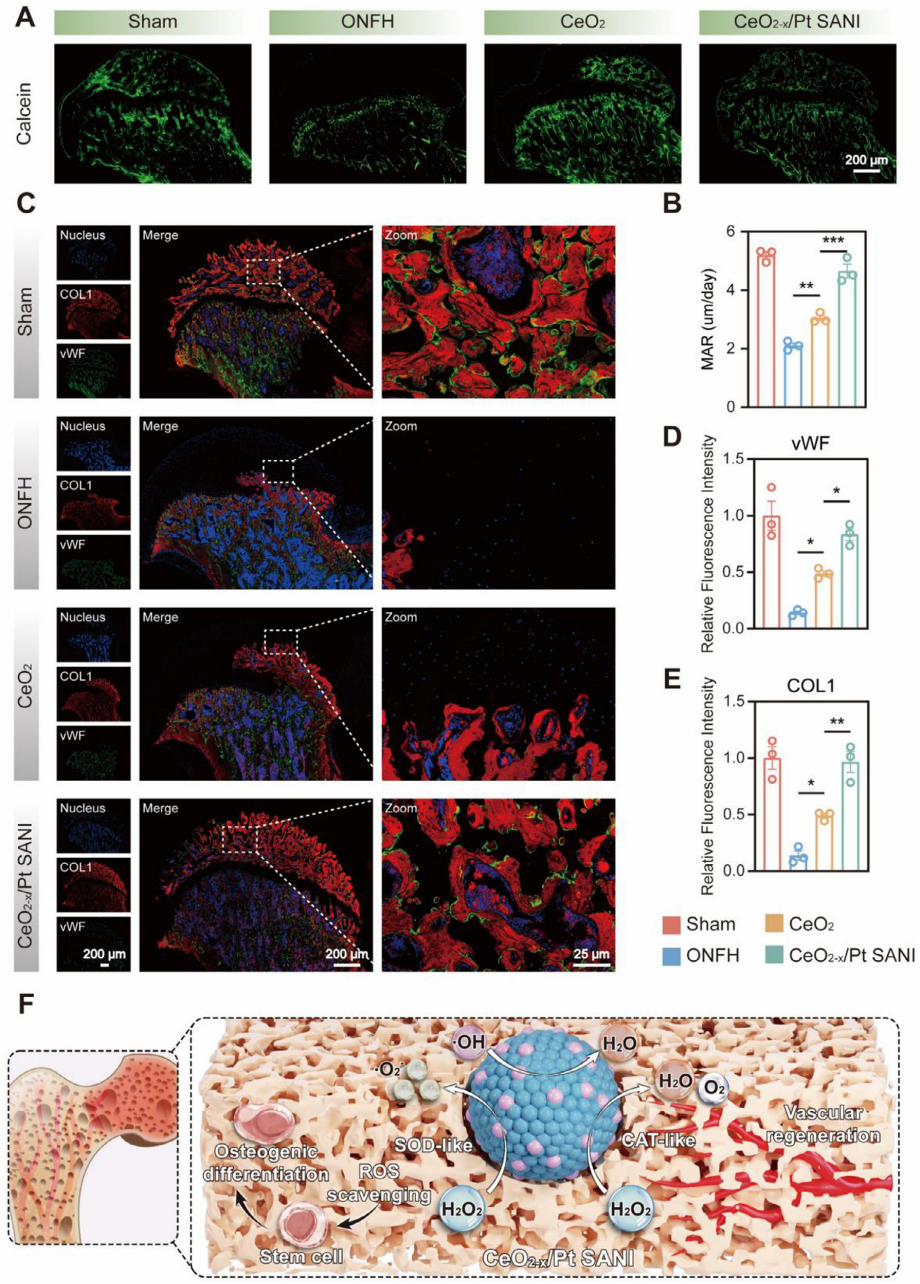
CeO_2‐x_/Pt SANI enhances mineralization and promotes coupled osteogenesis‐angiogenesis in vivo. (A) Representative calcein double‐labeling images showing new bone formation across groups. (B) Quantification of MAR indicating dynamic bone formation rate. (*n* = 3). (C) Immunofluorescence staining of nucleus (blue), COL1 (red) and vWF (green) in femoral head sections. Merged and magnified (Zoom) images highlight the spatial co‐localization and intensity differences. (D,E) Quantitative fluorescence intensity of vWF and COL1, respectively across treatment groups. (*n* = 3). (F) Schematic illustration of the in vivo mechanism for ROS elimination and vascular regeneration. Data are presented as mean ± SD. One‐way ANOVA with Tukey's post hoc test was used for multiple comparisons. *p < 0.05, **p < 0.01, ***p < 0.001.

To elucidate how CeO_2‐x_/Pt SANI facilitates bone repair at the tissue level, we assessed dynamic bone formation and the status of vascularization and matrix deposition in the treated femoral heads. Calcein double‐labeling revealed a marked reduction in the mineral apposition rate (MAR) in SONFH rats, indicative of compromised osteoblast activity and diminished bone formation. While CeO_2_ treatment alone partially restored MAR, CeO_2‐x_/Pt SANI achieved a significantly greater improvement compared to both the untreated SONFH and CeO_2_ groups (Figure 8A,B). This finding demonstrates that CeO_2‐x_/Pt SANI accelerates new bone mineral deposition in vivo, consistent with its enhanced support of osteogenesis observed in vitro. The superior improvement in MAR highlights the advantage of the Pt single‐atom catalyst in driving osteoblastic matrix synthesis.

In parallel, CeO_2‐x_/Pt SANI promoted revascularization and extracellular matrix formation in the regenerating femoral head. Immunofluorescence staining showed that SONFH lesions had markedly reduced expression of von Willebrand factor (vWF), an endothelial marker, and collagen type I (COL1), a principal bone matrix protein (Figure 8C). This reflects the poor vascularization and inferior matrix quality in untreated osteonecrotic bone. Quantification confirmed that the fluorescence intensities of vWF and COL1 in CeO_2‐x_/Pt SANI‐treated samples were significantly higher than those in untreated SONFH and CeO_2_‐treated group (Figure 8D,E). The elevated vWF indicates active neovascularization which improved blood supply, while the enhanced COL1 signifies abundant organized osteoid, pointing to high‐quality bone matrix formation. Notably, areas of vigorous neovascularization co‐localized with new collagen matrix in CeO_2‐x_/Pt SANI‐treated femoral heads, suggesting that angiogenesis and osteogenesis were tightly coupled during repair, a coupling often disrupted in SONFH due to oxidative damage.

These results underscore that CeO_2‐x_/Pt SANI creates a favorable microenvironment for coupled bone and blood vessel regeneration, consistent with relief of oxidative stress in the lesion. By persistently catalytic activity, the nanozyme preserved the functionality of osteoblasts and endothelial cells, enabling them to form new bone matrix and vasculature in tandem. This dual restoration of osteogenesis and angiogenesis is critical for effective bone healing, since bone formation and vascular supply are interdependent (Figure 8F). Moreover, the sustained catalytic activity provided by the Pt single‐atom design underpins the superior regenerative outcomes achieved with CeO_2‐x_/Pt SANI compared to CeO_2_ alone. In essence, this approach demonstrates that tuning nanomaterial properties (e.g., increasing oxygen‐vacancy concentration and optimizing electronic structure) to maintain redox homeostasis can rejuvenate the local tissue environment. In the case of CeO_2‐x_/Pt SANI, relieving the oxidative stress milieu of ONFH enabled normal bone repair and revascularization to proceed.

### 2.6 CeO_2‐x_/Pt SANI Reprograms the Bone Marrow Microenvironment in SONFH

**FIGURE 9:**
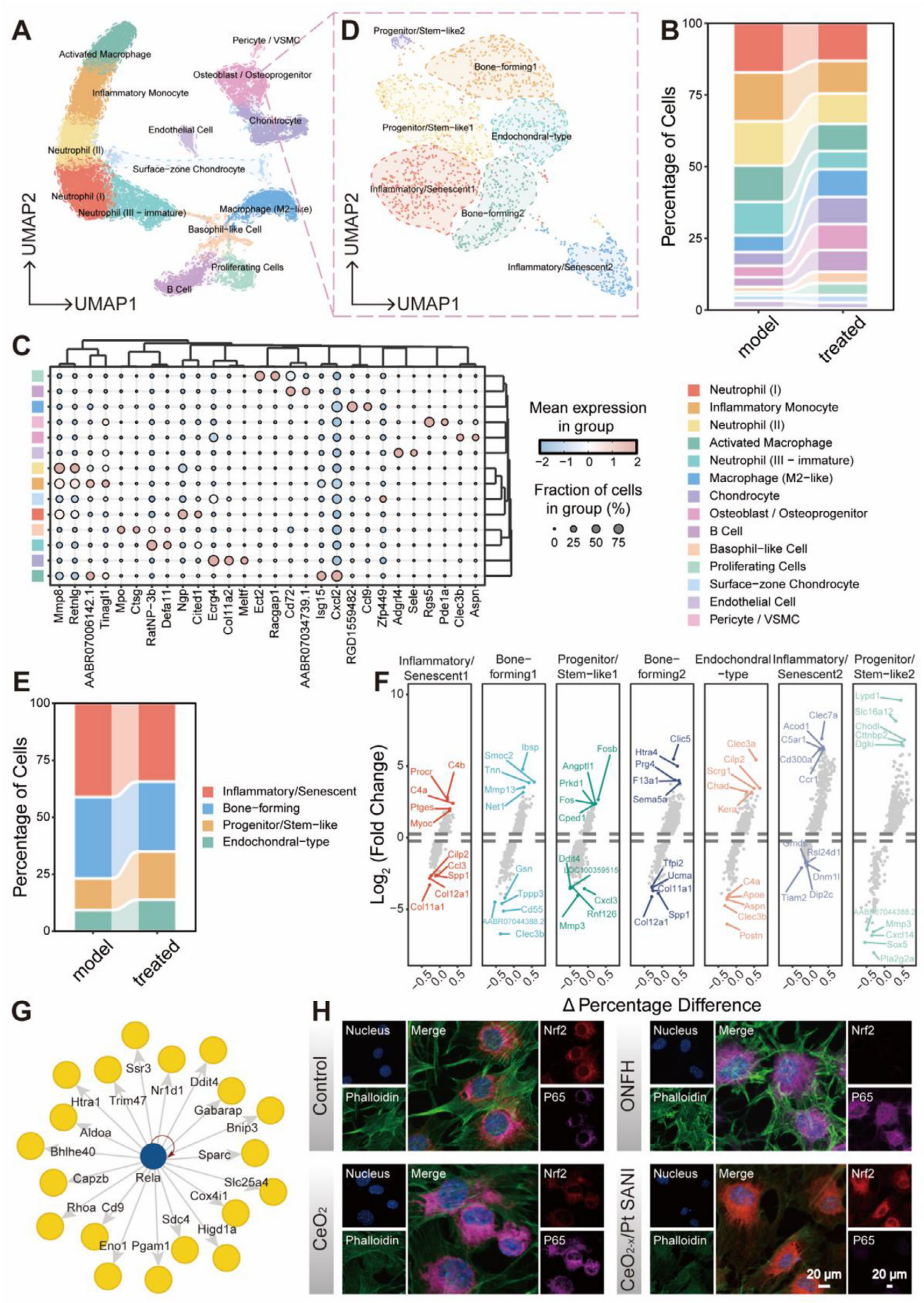
Single‐cell transcriptomics reveals that CeO_2‐x_/Pt SANI reprograms cellular composition and redox‐regulated signaling in SONFH. (A) UMAP visualization of single‐cell transcriptomes from femoral heads in SONFH and CeO_2‐x_/Pt SANI groups, identifying 16 major cell clusters. (B) Proportional distribution of major cell types, showing increased osteogenic and reduced inflammatory populations following CeO_2‐x_/Pt‐SANI treatment. (C) Dot plot of representative marker genes distinguishing inflammatory, osteogenic and stromal clusters. (D) Re‐clustering of osteo‐lineage cells into five subtypes including inflammatory/senescent, bone‐forming, progenitor/stem‐like and endochondral‐type. (E) Quantification of osteo‐lineage subtype proportions across SONFH and CeO_2‐x_/Pt SANI conditions. (F) Heatmap of top differentially expressed genes among osteogenic subtypes, illustrating enhanced matrix remodeling and suppressed inflammation in the treated group. (G) SCENIC‐inferred regulatory network highlighting key transcription factors (e.g., Rela and Trim47) enriched in SONFH and modulated by CeO_2‐x_/Pt SANI. (H) Immunofluorescence staining of HBMSCs showing increased nuclear translocation of Nrf2 (red) and reduced nuclear localization of p65 (purple) in CeO_2‐x_/Pt SANI ‐treated cells.

To further examine the impact of CeO_2‐x_/Pt SANI at a cellular level, we performed single‐cell RNA sequencing (scRNA‐seq) on femoral head marrow from SONFH model rats, comparing untreated with CeO_2‐x_/Pt SANI‐treated groups. The single‐cell analysis revealed that CeO_2‐x_/Pt SANI globally reprogrammed the bone marrow niche, enriching regenerative cell populations, suppressing inflammatory cells and modulating redox‐sensitive signaling pathways. Unsupervised clustering identified 16 distinct clusters, including osteoblasts/osteoprogenitors, endothelial cells and various immune cells (monocytes, macrophages, neutrophils and lymphocytes) among others (Figure 9A). Glucocorticoid‐induced SONFH severely skewed this cellular composition: inflammatory monocytes, neutrophils, and M1‐like (pro‐inflammatory, senescent) macrophages were overrepresented in SONFH samples, while osteogenic lineage cells and endothelial cells were greatly diminished compared to healthy tissue. This imbalance reflects a pathological microenvironment dominated by inflammation and impaired regeneration. Remarkably, CeO_2‐x_/Pt SANI treatment significantly reversed this imbalance (Figure 9B). Treated femoral heads contained a higher proportion of osteoprogenitor and endothelial cells and a lower proportion of pro‐inflammatory myeloid cells than untreated SONFH heads. For example, the osteoblastic lineage (osteoprogenitors and osteoblasts) was expanded in treated samples, whereas inflammatory monocytes and neutrophils were reduced. These shifts suggest that the nanozyme therapy created a more regenerative niche, likely by mitigating the oxidative/inflammatory milieu that would otherwise suppress reparative cells.

Distinct gene expression programs in rebalanced cell populations. Marker‐gene analysis illustrated how CeO_2‐x_/Pt SANI shifted marrow cell programs toward regeneration. As shown in Figure 9C, inflammatory myeloid clusters in SONFH expressed high levels of pro‐inflammatory and stress‐related genes, whereas osteoblastic and stromal clusters expressed osteogenic extracellular matrix genes, and endothelial clusters displayed angiogenic markers. CeO_2‐x_/Pt SANI treatment favored the latter programs over the former, consistent with a reduction in inflammatory signaling and enhancement of pro‐regenerative pathways. Focusing on the osteogenic lineage, reclustering of osteolineage cells identified several subpopulations, including an “inflammatory/senescent” osteoblast subset enriched in stress and cytokine genes, two subsets of active, matrix‐producing osteoblasts, and two subsets of osteoprogenitors with stem‐like, proliferative signatures (Figure 9D). In untreated SONFH, the osteolineage compartment was largely skewed toward the inflammatory/senescent state, with relatively few active bone‐forming cells or progenitors. CeO_2‐x_/Pt SANI treatment expanded the regenerative osteolineage subpopulations, with the proportions of progenitor/stem‐like osteoblasts and mature, bone‐forming osteoblasts increased at the expense of the inflammatory/senescent subset (Figure 9E and Figure S31). Differential expression analysis among these osteolineage subtypes confirmed the functional restoration of bone‐forming capacity with treatment. In CeO_2‐x_/Pt SANI‐treated samples, the active osteoblast clusters upregulated osteogenic and matrix‐synthesis genes, while the inflammatory osteoblast subset downregulated stress‐ and cytokine‐related genes (Figure 9F; Figures S32 and S33A). This indicates that CeO_2‐x_/Pt SANI not only rebalances the abundance of cell types but also resets their gene expression programs to ones conducive for bone repair.

We next investigated upstream regulators that might drive these transcriptional shifts. Single‐cell regulatory network inference and clustering (SCENIC) analysis suggested that SONFH samples were enriched for transcription factors associated with oxidative stress and inflammation such as Rela, the p65 subunit of NF‐κB and Trim47 (Figure 9G). With CeO_2‐x_/Pt SANI treatment, the activity of such pro‐inflammatory factors was diminished. Interestingly, the canonical antioxidant response regulator Nrf2 was not among the top differentially active factors in SCENIC, but we observed increased expression of several Nrf2 target genes such as SOD3 in CeO_2‐x_/Pt SANI–treated cells, suggesting an indirect enhancement of Nrf2 signaling (Figure S33B). To validate these findings at the protein level, we performed immunofluorescence for Nrf2 and NF‐κB in HBMSCs under SONFH‐mimetic conditions. CeO_2‐x_/Pt SANI‐treated cells showed robust nuclear translocation of Nrf2 and a clear reduction in nuclear NF‐κB p65, relative to untreated cells (Figure 9H). This supports the notion that CeO_2‐x_/Pt SANI alleviates NF‐κB‐driven inflammatory signaling while bolstering antioxidant defenses in oxidatively stressed bone cells. Collectively, the scRNA‐seq and molecular data demonstrate that CeO_2‐x_/Pt SANI therapy fundamentally rewires the pathological bone marrow niche in SONFH. It suppresses inflammatory circuits and rejuvenates osteogenic and angiogenic programs, thereby reestablishing redox homeostasis and enabling tissue regeneration. These results illustrate the potential of atomically engineered nanozymes to achieve “microenvironmental reprogramming”, essentially shifting a diseased cellular milieu back toward a healthy, regenerative state.

### 2.7 CeO_2‐x_/Pt SANI Restores Mitochondrial Homeostasis and Alleviates Oxidative Stress

**FIGURE 10:**
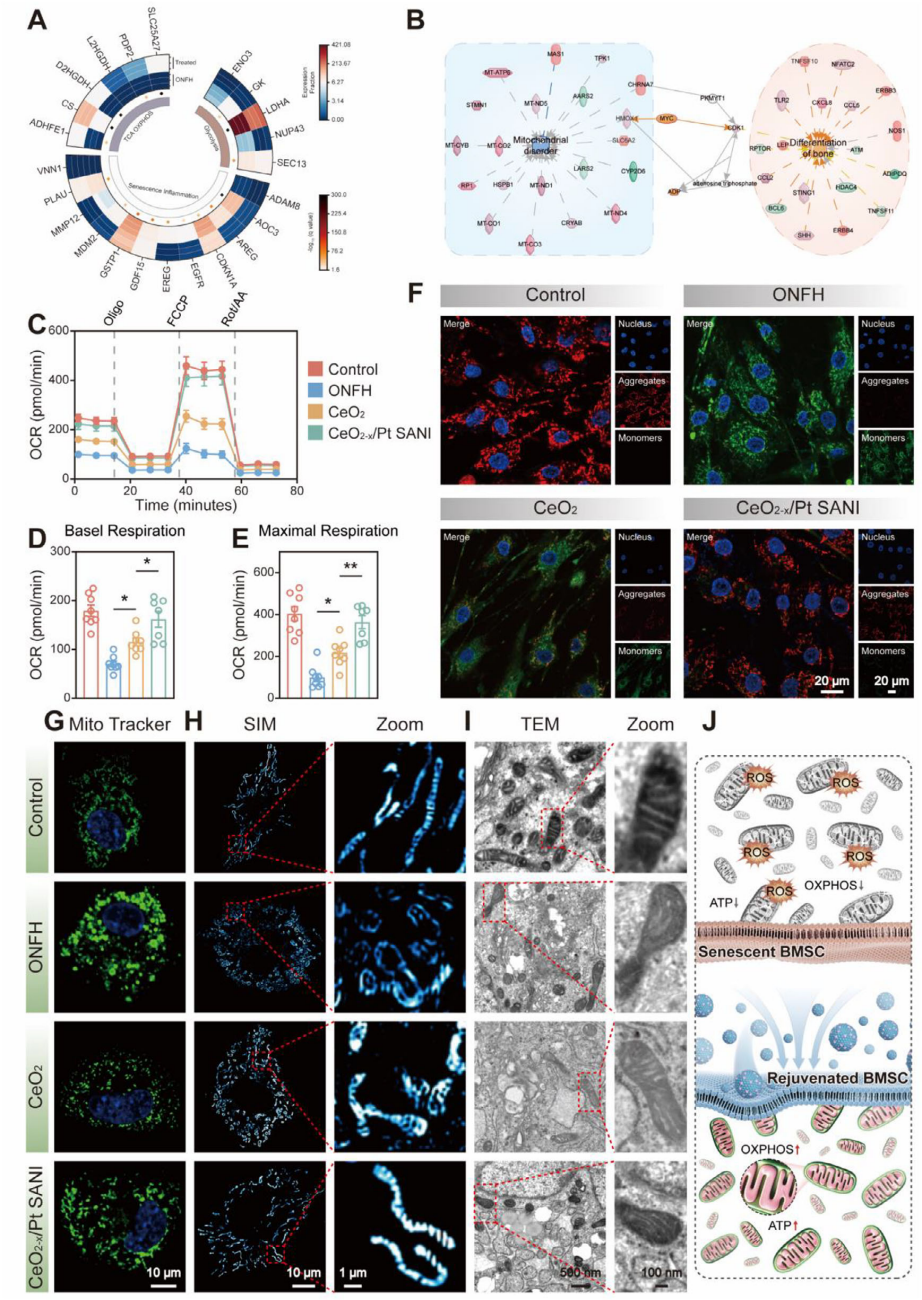
CeO_2‐x_/Pt SANI restores mitochondrial function and proteostasis in SONFH. (A) Transcriptomic heatmap of mitochondrial‐associated gene sets showing downregulation of OXPHOS, TCA cycle and translation‐related genes in SONFH, reversed by CeO_2‐x_/Pt SANI. (B) IPA identifying “mitochondrial disorder” and oxidative metabolism disruption as top altered pathways in SONFH. (C) OCR kinetics measured by Seahorse assay across four groups. (*n* = 3). (D,E) Quantification of basal respiration and maximal respiration. (*n* = 3). (F) Immunofluorescence staining of JC‐1 aggregates and monomers distribution in each group. (G) Mito‐Tracker staining of mitochondrial networks. (H) SIM revealing mitochondrial morphology. right panels show zoomed‐in mitochondrial tubules. (I) TEM images showing mitochondrial ultrastructure, with red boxes indicating zoomed regions. (J) Schematic illustration of CeO_2‐x_/Pt SANI‐mediated protection against oxidative damage via ROS clearance, mitochondrial preservation and stem cell rejuvenation. Data are presented as mean ± SD. One‐way ANOVA with Tukey's post hoc test was used for multiple comparisons. *p < 0.05, **p < 0.01.

Because oxidative injury often targets intracellular organelles, we examined whether CeO_2‐x_/Pt SANI protects mitochondrial bioenergetics and protein homeostasis in SONFH. Transcriptomic profiling revealed that SONFH caused broad downregulation of genes involved in mitochondrial metabolism and protein synthesis. Key energy‐production pathways, including oxidative phosphorylation (OXPHOS), the tricarboxylic acid (TCA) cycle and fatty acid β‐oxidation, were suppressed in SONFH, as was the expression of many ribosomal proteins and translation factors (Figure 10A). This indicates a collapse of mitochondrial energy generation and proteostatic capacity under chronic oxidative stress. Ingenuity Pathway Analysis reinforced this, highlighting “mitochondrial dysfunction” as a top altered pathway in SONFH, along with disrupted oxidative metabolism and impaired protein synthesis (Figure 10B). Notably, CeO_2‐x_/Pt SANI treatment partially rescued these transcriptional changes, restoring the expression of numerous OXPHOS and other metabolic genes toward normal levels. In essence, CeO_2‐x_/Pt SANI helped sustain the transcription of genes for energy metabolism and protein homeostasis that were otherwise repressed by glucocorticoid stress.

Beyond these specific pathways, global enrichment analyses underscored the same trends (Figure S33C–E). KEGG analysis showed that ONFH downregulated oxidative metabolism, extracellular matrix‐receptor interactions and ribosome biogenesis, while upregulating pathways related to advanced glycation end‐products (AGE–RAGE signaling) and adipogenesis (PPAR signaling). CeO_2‐x_/Pt SANI counteracted these changes, enriching processes associated with ATP production, extracellular matrix organization and cytoskeletal regulation. Together, these results indicate that CeO_2‐x_/Pt SANI not only safeguards mitochondrial energy metabolism but also broadly supports protein synthesis, matrix maintenance and cellular structural integrity under oxidative stress, effectively preserving the cellular infrastructure needed for bone regeneration.

We next measured mitochondrial respiration to functionally validate the transcriptomic findings. Using Seahorse extracellular flux analysis, we found that dexamethasone‐treated (SONFH‐mimic) HBMSCs had dramatically reduced mitochondrial oxygen consumption rates (OCR) compared to healthy controls. Both basal respiration and maximal respiratory capacity were significantly lower in SONFH cells, confirming compromised OXPHOS function. CeO_2‐x_/Pt SANI treatment markedly improved cellular respiration. Treated cells exhibited OCR traces nearly overlapping with healthy controls, with substantial increases in basal OCR and restoration of maximal respiration compared to untreated SONFH cells. Quantitatively, CeO_2‐x_/Pt SANI rescued basal respiration to ≈90% of control levels and approximately doubled the maximal OCR relative to the untreated SONFH group. By contrast, CeO_2_ nanoparticles produced only a moderate improvement (Figure 10C–E). Consistent with these results, direct measurement of intracellular ATP showed that glucocorticoid exposure severely depleted ATP levels, whereas CeO_2‐x_/Pt SANI significantly restored cellular ATP to near‐normal levels (Figure S34). Thus, CeO_2‐x_/Pt SANI effectively preserves mitochondrial ATP‐generating capacity under oxidative insult, likely by preventing ROS‐induced mitochondrial damage. This improvement in bioenergetics is critical for bone cells, as osteogenic differentiation and matrix production are energy‐intensive processes. To evaluate mitochondrial membrane potential (ΔΨm), we used the JC‐1 probe in HBMSCs under SONFH‐mimetic stress. In healthy control cells, strong red fluorescence indicated well‐polarized mitochondria with high ΔΨm. In contrast, SONFH‐treated cells showed predominantly green fluorescence, reflecting mitochondrial depolarization. CeO_2‐x_/Pt SANI‐treated cells restored the red/green ratio to near‐control levels, with pronounced red puncta indicating intact membrane potential and preserved mitochondrial function (Figure 10F).

High‐resolution imaging provided direct visual confirmation of organelle‐level protection by CeO_2‐x_/Pt SANI. In healthy control cells, mitochondria formed extensive, tubular networks across the cytoplasm, as revealed by confocal imaging with MitoTracker and structured illumination microscopy (SIM). Under SONFH conditions, this mitochondrial network became fragmented and punctate, with significantly shortened tubules and disorganized distribution, hallmarks of mitochondrial stress due to excess ROS and disrupted fission‐fusion dynamics. Strikingly, CeO_2‐x_/Pt SANI preserved a healthy mitochondrial morphology despite the glucocorticoid stress, with treated cells retaining interconnected, elongated mitochondrial networks comparable to those in untreated controls (Figure 10G,H). Ultrastructural examination by TEM reinforced these findings. Control cells showed mitochondria with intact double membranes and well‐defined cristae, whereas SONFH cells contained swollen, vacuolated mitochondria with ruptured or collapsed cristae. In CeO_2‐x_/Pt SANI‐treated cells, mitochondrial ultrastructure was largely normal, with clearly delineated membranes and cristae (Figure 10I). This preservation of structure aligns with the functional OCR data, confirming that CeO_2‐x_/Pt SANI safeguards mitochondrial integrity and function under oxidative challenge.

Based on these findings, we propose a comprehensive mechanism by which CeO_2‐x_/Pt SANI confers cytoprotection and drives tissue regeneration in SONFH (Figure 10J). CeO_2‐x_/Pt SANI acts as a potent catalytic antioxidant that continuously eliminates excess ROS in the bone microenvironment. By doing so, it prevents ROS‐mediated damage to critical cellular components, particularly mitochondria. Preserving mitochondrial bioenergetics ensures that bone cells (especially osteoprogenitors and osteoblasts) have sufficient ATP and metabolic support for new bone formation. Beyond these cell‐intrinsic protections, redox modulation of CeO_2‐x_/Pt SANI also rebalances cellular signaling, dampening NF‐κB‐driven inflammatory pathways and indirectly enhancing Nrf2‐mediated antioxidant responses, which allows proper osteogenic and angiogenic gene programs to proceed. The net result is a rejuvenation of stem/stromal cell function and an overall enhancement of the bone's regenerative capacity. Notably, unlike conventional antioxidants that act stoichiometrically and have transient effects, CeO_2‐x_/Pt SANI provides continuous catalytic relief from oxidative stress, offering a sustained therapeutic benefit.

## 3 Conclusions

Overall, our results demonstrate that a rationally designed single‐atom nanozyme (CeO_2‐x_/Pt SANI) can counteract multiple pathological processes in an oxidative stress‐induced bone disorder. Through persistent redox modulation, CeO_2‐x_/Pt SANI reversed cellular senescence and adipogenic degeneration, enhanced osteogenic differentiation and angiogenesis, rebalanced the inflammatory microenvironment and preserved mitochondrial function and protein homeostasis. Collectively, these multifaceted effects led to robust bone regeneration and the recovery of weight‐bearing function in our SONFH animal model. These findings underscore the translational potential of CeO_2‐x_/Pt SANI for clinical SONFH therapy. Moreover, this work highlights the broader therapeutic promise of targeting oxidative stress with advanced nanozyme catalysts. It also underscores the importance of microenvironmental reprogramming in the treatment of degenerative diseases characterized by oxidative damage. By integrating materials science and bone biology, our approach paves the way for next‐generation antioxidant therapies that are both potent and durable in restoring tissue health.

## 4 Methods

### 4.1 Chemicals

2,2’‐azino‐bis(3‐ethylbenzothiazoline‐6‐sulfonic acid) diammonium salt (ABTS), hydrogen peroxide (H_2_O_2_), Hexahydrate cerium(IV) nitrate (Ce(NO_3_)_2_•6H_2_O), acetic acid, ethylene glycol, potassium tetrachloroplatinate (II) (K_2_PtCl_4_), and ethanol were purchased from Sinopharm Chemical Reagents (Shanghai, China). 3,3’,5,5’‐tetramethylbenzidine (TMB), titanous sulfate (Ti(SO_4_)_2_) and ferric chloride (FeCl_3_) were provided by Sigma–Aldrich (St. Louis, USA). ROS assay kit for superoxide anion (S0064S, Beyotime), JC‐1 (M8650, Solarbio), Calcein/PI kit (C2015S, Beyotime), Mito‐Tracker red CMXRos (C1035, Beyotime), Lyso‐Tracker Green (C1047S, Beyotime).

### 4.2 Instruments

Powder X‐ray diffraction (XRD) patterns were recorded on a Rigaku Miniflex‐600 diffractometer. Transmission electron microscope (TEM) images were taken by Hitachi‐7700. High‐angle annular dark field scanning transmission electron microscopy (HAADF‐STEM) images were recorded by JEM‐ARM200F (JEOL) TEM/STEM with a spherical aberration corrector. The energy‐dispersive X‐ray spectroscopy (EDS) mapping was performed by JEM‐2100F. X‐ray photoelectron spectroscopy (XPS) spectra were collected on scanning X‐ray microprobe (PHI 5000 Verasa, ULAC‐PHI). Scanning electron microscopy (SEM) images were taken by Nova NanoSEM 230. Fluorescence imaging was performed by confocal microscopy (Nikon C2). The absorption spectra were measured by a ultraviolet–visible (UV–vis) UH4150 spectrophotometer (Hitachi). Metal content was measured by using inductively coupled plasma mass spectrometer (ICP‐MS, PlasmaQuad 3, Thermo Elemental). Hydrodynamic diameters and zeta potentials were determined by a Zetasizer nano ZS instrument (Malvern).

## 5 Experimental Section

### 5.1 Synthesis of CeO_2_ and CeO_2‐x_/Pt SANI

To synthesize CeO_2_, 1 g Ce(NO_3_)_2_•6H_2_O, 1 mL H_2_O, and 1 mL acetic acid were dissolved in 25 mL ethylene glycol. The mixture was stirred for 30 min to ensure complete dissolution and homogeneity. Subsequently, the mixture was transferred into a high‐pressure hydrothermal reaction vessel and heated at 180°C for 200 min. After cooling, the CeO_2_ product was collected by centrifugation, washed several times with water and methanol to remove any residual reactants, and dried at 65°C in a vacuum oven overnight.

For the synthesis of CeO_2‐x_/Pt SANI, as prepared 200 mg of CeO_2_, and 5 mg of K_2_PtCl_4_ were dissolved in 30 mL of H_2_O in a 50 mL flask and the mixture was stirred for 12 h to achieve the effective adsorption of Pt precursors by CeO_2_. Then the CeO_2_@Pt product was collected by centrifugation, washed several times with water and methanol to remove any unreacted precursors, and dried at 65°C in a vacuum oven overnight. Finally, the CeO_2_@Pt product was placed in a tubular furnace and pyrolyzed at 250°C for 2 h under a 10% H_2_/Ar atmosphere to achieve the desired nanostructure and enhance the catalytic properties.

### 5.2 Detection of •OH Scavenging Activity

Hydroxyl radicals were generated using the Fenton reaction with FeSO_4_·7H_2_O and H_2_O_2_. In a 96‐well plate, the following reagents were added sequentially: 830 uL buffer, 50 µL of FeSO_4_·7H_2_O (1.8 mM), 100 µL of CeO_2_ or CeO_2‐x_/Pt SANI (100 µg mL^−1^), 10 µL of TMB (4 mg mL^−1^), and 10 µL of H_2_O_2_ (1 mM) in pH 7.4. The mixture was incubated at 37°C for 30 min in the dark. The absorbance was measured at wavelengths ranging from 550 to 750 nm using a microplate reader to quantify the •OH elimination.

To confirm the •OH scavenging capability of CeO_2_ and CeO_2‐x_/Pt SANI, ESR analysis was conducted using DMPO as the spin trap. A mixture containing 100 µg mL^−1^ CeO_2_ or CeO_2‐x_/Pt SANI, 10 uM FeCl_3_, 10 mM H_2_O_2_, and 100 mM DMPO in pH 7.4 was prepared. This mixture was then transferred to a quartz tube for ESR measurement to assess the scavenging efficiency of the CeO_2_ and CeO_2‐x_/Pt SANI against •OH.

### 5.3 ABTS‐Like Free Radical Scavenging Activity

The free radical scavenging capacity of CeO_2_ and CeO_2‐x_/Pt SANI was evaluated using the discoloration test of ABTS radical cations (ABTS•^+^). ABTS•^+^ was generated by mixing 7 mL of ABTS aqueous solution with 1 mM FeCl_3_ and allowing the mixture to react for 10 min. Different concentrations of CeO_2_ or CeO_2‐x_/Pt SANI (0–100 µg mL^−1^) were then added to the ABTS•^+^ solution. Absorbance was measured at wavelengths ranging from 500 to 750 nm to assess the radical scavenging activity.

### 5.4 SOD‐Like Activity of CeO_2_ and CeO_2‐x_/Pt SANI

To evaluate the SOD‐like activity of CeO_2_ and CeO_2‐x_/Pt SANI, various concentrations (0–100 µg mL^−1^) were prepared in an assay buffer. In a 96‐well plate, 20 µL of each sample was mixed with 200 µL of WST working solution. The reaction was initiated by adding 20 µL of enzyme working solution, followed by incubation at 37°C for 30 min. The absorbance was measured at wavelengths ranging from 350 to 525 nm using a microplate reader.

To confirm the •O_2_^−^ scavenging capability of CeO_2_ and CeO_2‐x_/Pt SANI, ESR analysis was conducted using DMPO as the spin trap. A mixture containing 100 µg mL^−1^ CeO_2_ or CeO_2‐x_/Pt SANI, 10 mM xanthine oxidase, 100 mM xanthine, and 100 mM DMPO was prepared. This mixture was then transferred to a quartz tube for ESR measurement to assess the scavenging efficiency of the CeO_2_ and CeO_2‐x_/Pt SANI against •O_2_^−^.

### 5.5 CAT‐Like Activity of CeO_2_ and CeO_2‐x_/Pt SANI

The CAT‐like activity of CeO_2_ and CeO_2‐x_/Pt SANI were assessed by measuring oxygen generation using a dissolved oxygen analyzer. A solution of 20 mM H_2_O_2_ was prepared in PBS buffer solution. To each buffer solution, 100 ug mL^−1^ CeO_2_ or CeO_2‐x_/Pt SANI was added. The oxygen concentration was immediately detected, and data were continuously recorded for 5 min to assess the CAT‐like activity.

### 5.6 DFT Calculations

All density functional theory (DFT) calculations were performed using the Vienna Ab Initio Simulation Package (VASP) [44]. The exchange‐correlation functional was described within the generalized gradient approximation (GGA) as parameterized by Perdew–Burke–Ernzerhof (PBE). Projected augmented wave (PAW) potentials were adopted to represent the core electrons, while valence electrons were expanded in a plane‐wave basis set with a kinetic energy cutoff of 450 eV. To treat partial orbital occupancies, Gaussian smearing with a width of 0.05 eV was applied. The self‐consistent electronic iteration was converged when the energy difference fell below 10^−^^5^ eV, and geometric relaxation was terminated once the residual forces were less than 0.05 eV/Å. Dispersion corrections were included via Grimme's DFT‐D3 scheme. A vacuum layer of 20 Å was inserted perpendicular to the slab surface to avoid interlayer interactions. The Brillouin zone was sampled using a 2 × 2 × 1 Monkhorst–Pack mesh for surface models. Adsorption energy (Eads) was defined as Eads = Ead/sub − Ead − Esub, where Ead/sub, Ead, and Esub correspond to the total energies of the adsorbate–substrate system, isolated adsorbate, and clean substrate, respectively. The Gibbs free energy (G) was computed as G = Eads + ZPE − TS, where ZPE and TS denote the zero‐point energy and entropic contribution, respectively.

### 5.7 Senescence‐Associated β‐Galactosidase (SA‐β‐gal) Staining

BMSCs were seeded into 12‐well culture plates and maintained until reaching approximately 70%–80% confluence. Cellular senescence was then assessed using a β‐galactosidase Staining Kit (Solarbio, China, G1580). After two washes with PBS, the cells were fixed and subsequently incubated with the β‐Gal staining solution at 37°C overnight. Following another PBS rinse, the stained cells were examined under a light microscope.

### 5.8 CCK‐8 Assay

Cell viability was determined using the Cell Counting Kit‐8 (CCK‐8) assay. BMSCs cells were treated with varying concentrations of H_2_O_2_, CeO_2‐x_/Pt SANI CeO_2_ and for 24 h. Subsequently, CCK‐8 reagent was added to each well, and the absorbance at 450 nm was measured using a microplate reader after further incubation.

### 5.9 Oil Red O Staining

Adipogenic differentiation was induced for 14 days using a commercial adipogenic medium (Cyagen, China, HUXMX‐90031). Cells were fixed in 4% paraformaldehyde for 30 min, washed, and stained with freshly prepared Oil Red O solution (0.3% in isopropanol) for 20 min‐stained lipid droplets were imaged under an inverted microscope.

### 5.10 Alizarin Red S (ARS) Staining

BMSCs were induced in osteogenic differentiation medium for 14 days. Cells were fixed and stained with 2% ARS solution (Cyagen, China, HUXMX‐90021) for 30 min. After washing, calcium nodules were visualized under light microscopy.

### 5.11 Alkaline Phosphatase (ALP) Staining

Early osteogenic differentiation was evaluated after 7 days of induction. Cells were washed with PBS, fixed, and stained using the BCIP/NBT Alkaline Phosphatase Color Development Kit (Beyotime, China, C3206).

### 5.12 Quantitative Reverse Transcription Polymerase Chain Reaction (qRT‐PCR)

Total RNA was extracted using TRIzol reagent (Invitrogen, USA, 15596018CN). One microgram of RNA was reverse‐transcribed using the PrimeScript RT reagent Kit (Takara, China, RR047A). qRT‐PCR was performed using TB Green Premix Ex Taq (Takara, China, RR420A) on a QuantStudio 5 Real‐Time PCR System. Relative expression levels were calculated using the 2^‐ΔΔCt method and normalized to GAPDH. Primer sequences are listed in Table S3.

### 5.13 Reactive Oxygen Species (DCFH‐DA) Detection

Total intracellular ROS levels were assessed using the fluorescent probe 2',7'‐dichlorodihydrofluorescein diacetate (DCFH‐DA). Treated cells were co‐stained with DCFH‐DA (10 µM) and Hoechst 33342 (20 µM), and the green fluorescence intensity was monitored by confocal microscopy.

### 5.14 Tube Formation Assay

Matrigel (Corning, USA, 354277) was thawed on ice and added (10 mg mL^−1^) to pre‐chilled 96‐well plates, polymerized at 37°C for 30 min. HUVECs (2 × 10^4^ cells well^−1^) were seeded with conditioned medium containing CeO_2_, CeO_2‐x_/Pt SANI or control supernatant. After 6 h incubation, tube‐like structures were imaged under an inverted microscope. Parameters (tube junctions and node number) were quantified using ImageJ with the Angiogenesis Analyzer plugin.

### 5.15 Transwell Migration Assay

HUVECs (1 × 10^5^ cells well^−1^) were seeded in the upper chamber (8 µm pores, Corning, USA, 3422) in serum‐free medium. The lower chamber contained ECM supplemented with 10% FBS and conditioned medium. After 12 h incubation, migrated cells on the lower membrane surface were fixed with 4% paraformaldehyde, stained with crystal violet, and counted from five random fields.

### 5.16 Wound Healing Assay

HUVECs were seeded to reach 90% confluence in 6‐well plates. A linear scratch was made using a sterile 200 µL pipette tip. Detached cells were removed, and fresh medium was added. Images were taken at 0 and 24 h. Migration rate was determined by the reduction in wound width relative to the initial width using ImageJ.

### 5.17 Micro‐CT Scanning

After euthanasia, femoral heads were fixed in 4% paraformaldehyde and scanned using a high‐resolution micro‐CT system (Siemens) at 50 kV, 500 µA and 9 µm resolution. Morphometric parameters (BV/TV, Tb.N, Tb.Th, Tb.Sp) were analyzed with CTAn software.

### 5.18 Gait Analysis

Motor function was assessed using DigiGait treadmill system (Mouse Specifics, Inc., USA). Rats were trained for three consecutive days before testing. Walking patterns were recorded at a constant speed of 15 cm s^−1^. Parameters including stride length, stance time, stride frequency and swing time were averaged from three consistent runs per animal.

### 5.19 Histological Staining

Decalcified femoral heads were paraffin‐embedded and sectioned at 5 µm thickness. Sections were stained with hematoxylin and eosin (H&E) to evaluate bone architecture and with Masson's trichrome for collagen deposition. Slides were scanned using a digital slide scanner (3D HISTECH), and quantitative analysis was performed using CaseViewer software.

### 5.20 Dynamic Bone Formation Labeling

Calcein double labeling was performed to determine bone formation rates. Rats were intraperitoneally injected with calcein (20 mg kg^−1^; Sigma–Aldrich) on days 10 and 3 before sacrifice. Undecalcified sections were prepared using polymethyl methacrylate (PMMA) embedding, and the distance between two fluorescent labels was measured using ImageJ. The mineral apposition rate (MAR) was calculated as distance/time (µm/day).

### 5.21 Immunofluorescence Staining

To assess the subcellular localization and expression levels of target proteins, immunofluorescence staining was performed. Briefly, treated cells were fixed with 4% paraformaldehyde for 15 min at room temperature and then permeabilized with 0.1% Triton X‐100 for 10 min. After blocking with 5% bovine serum albumin (BSA) for 1 h to prevent non‐specific binding, the cells were incubated overnight at 4°C with primary antibodies against primary antibodies against COL1A1 (1:100, Abcam) or vWF (1:100, Abcam) diluted in blocking buffer. Following extensive washing with PBS, the cells were incubated with appropriate fluorophore‐conjugated secondary antibodies (Alexa Fluor 488 or 594) for 1 h at room temperature in the dark. Nuclei were counterstained with DAPI. Finally, the samples were mounted with an anti‐fade mounting medium. Fluorescence images were captured using a confocal laser scanning microscope, and the fluorescence intensity was quantified using ImageJ software.

### 5.22 Single‐Cell RNA Sequencing (scRNA‐seq)

Femoral heads were enzymatically dissociated with collagenase type I (2 mg mL^−1^) and DNase I (100 U mL^−1^) for 45 min at 37°C. Cells were filtered (70 µm) and loaded into the 10x Genomics Chromium Controller. Libraries were prepared using the Single Cell 3’ v3.1 kit and sequenced on Illumina NovaSeq 6000. Data were processed with Cell Ranger (v6.0.1) and analyzed in Seurat (v4.1). Clusters were visualized by UMAP, and marker genes were identified using Wilcoxon rank‐sum test (*p* < 0.05).

### 5.23 RNA Sequencing (Bulk RNA‐seq)

Total RNA was extracted and subjected to mRNA enrichment using oligo(dT) beads. Libraries were constructed using NEBNext Ultra RNA Library Prep Kit and sequenced (2 × 150 bp) on NovaSeq 6000. Reads were aligned to Rnor_6.0 genome using HISAT2, quantified with featureCounts, and analyzed by DESeq2. Differentially expressed genes were defined as |log_2_FC| > 1, *p* < 0.05. GO and KEGG enrichment analyses were conducted using ClusterProfiler.

### 5.24 Seahorse XF Mitochondrial Stress Test

Oxygen consumption rate (OCR) was measured using the Seahorse XF96 Analyzer (Agilent Technologies, USA, 102416‐100). BMSCs were seeded (2 × 10^4^ cells/well) and treated with Pt‐based nanomaterials for 48 h. The Mito Stress Test kit was used to sequentially inject oligomycin, FCCP and rotenone/antimycin A. Basal respiration and maximal respiration were calculated using Wave software.

### 5.25 JC‐1 Mitochondrial Membrane Potential Assay

Changes in MMP were assessed using the JC‐1 fluorescent probe (Yisheng, China, 40706ES60). Following the manufacturer's instructions, treated cells were stained with JC‐1, and the shift in fluorescence from red (aggregates) to green (monomers) was observed. The ratio of red to green fluorescence intensity, indicative of MMP depolarization, was calculated.

### 5.26 MitoTracker and Structured Illumination Microscopy

For confocal laser scanning microscopy, BMSCs were stained with MitoTracker Green FM (Yisheng, China, 40742ES50), while PK Mito Deep Red (Warbio, China, PKMDR‐1) was used for structured illumination microscopy (SIM).

### 5.27 TEM

Cells were fixed with 2.5% glutaraldehyde, post‐fixed with 1% osmium tetroxide, dehydrated and embedded in epoxy resin. Ultrathin sections (70 nm) were stained with uranyl acetate and lead citrate. Images were captured using a TEM to evaluate mitochondrial integrity, cristae structure and matrix density.

### 5.28 Enzyme‐Linked Immunosorbent Assay

The levels of cytokines in cell culture supernatants or mouse plasma were quantified using commercial enzyme‐linked immunosorbent assay (ELISA) kits (Cloud‐Clone Corp.) according to the manufacturer's instructions. Absorbance was measured using a SpectraMax i3x microplate reader (Molecular Devices).

### 5.29 Statistical Analysis

Data analysis was performed with GraphPad Prism, Origin, VASP, Avantage, and Image J software, while IVIS was utilized for in vivo imaging processing. Results from at least three independent replicates are expressed as mean ± standard deviation (SD). Details regarding sample size (*n* ≥ 3), normalization methods, and the specific statistical tests applied are provided in the respective figure legends. To assess significant variations between the control and experimental groups, one‐way analysis of variance (ANOVA) with a Tukey post‐hoc test were employed. These analyses were conducted using Prism 10.4 software, and differences were considered statistically significant at *p* < 0.05 (represented as *p < 0.05, **p < 0.01, ***p < 0.001, or not significant (ns)).

## Author Contributions

**Y. Z**., **Z. L**., and **X. C**., and **D. W**. designed the research. **Y. Z**., **Z. W**., **R. W**., and **P. W**. performed the research. **Y. Z**., **Z. L**., **X. C**., and **P. W**. analyzed the data. **Y. Z**., **Y. W**., **X. Y**., **Y. B**., and **J. X**. conceived the manuscript. **X. W**. and **L. W**. supervised the research.

## Declarations

Animal experiments were conducted in accordance with the protocol approved by the Institutional Animal Care and Use Committee of Fujian Medical University (IACUC FJMU 2022‐0608).

## Formal Statement

We hereby formally declare that no AI‑based tools or artificial intelligence technologies were employed at any stage during the preparation of this manuscript, including but not limited to text generation, language polishing, figure creation, data processing, or data analysis. All work was completed by the authors through independent research and writing.

## Conflicts of Interest

The authors declare no conflicts of interest.

## Supporting information




**Supporting File**: advs75840‐sup‐0001‐SuppMat.docx.

## Data Availability

The data that supports the findings of this study are available in the supplementary material of this article.
