# Supplementary material for: Oxygen Vacancy‐Enriched Platinum Single‐Atom Nanozyme Wrapped in Nanoislands: Unlocking Catalytic Activity and Reprogramming Redox Microenvironment for Osteonecrosis Repair
Source: Adv Sci (Weinh). 2026 May 26:e75840. Online ahead of print. doi: 10.1002/advs.75840 (PMC13336132; doi:10.1002/advs.75840)
Supplement: Supplementary file 1 — Supporting File: advs75840‐sup‐0001‐SuppMat.docx. [file ADVS-9999-e75840-s001.docx]

**Oxygen Vacancy-Enriched Platinum Single-Atom Nanozyme Wrapped in Nanoislands: Unlocking Catalytic Activity and Reprogramming Redox Microenvironment for Osteonecrosis Repair**


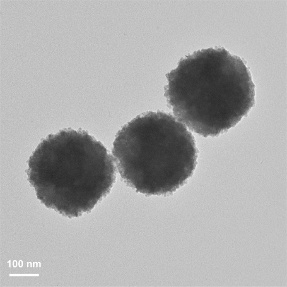


**Fig. S1. The TEM image of CeO_2_.**

**
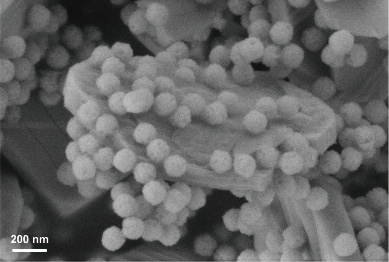
**

**Fig. S2. The SEM image of CeO_2_.**


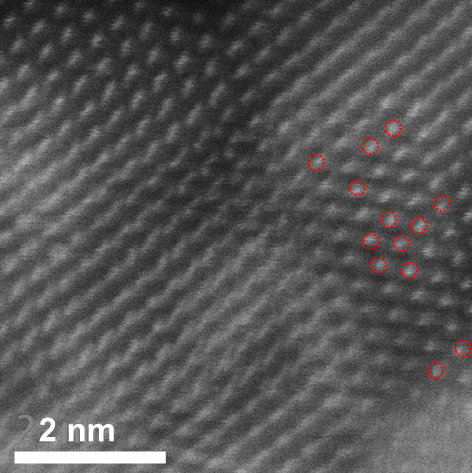

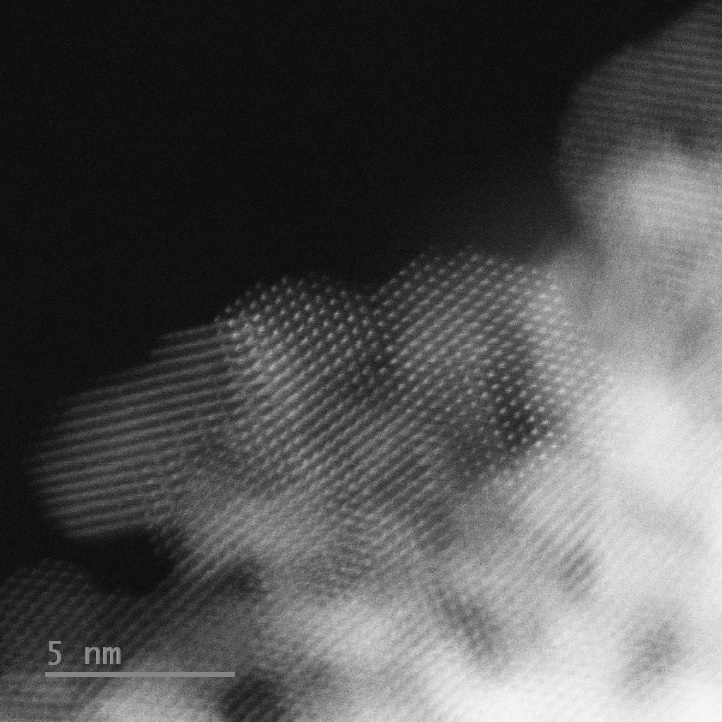


**Fig. S3. The high- resolution and low-resolution HAADF-STEM image of CeO_2-x_/Pt SANI.**


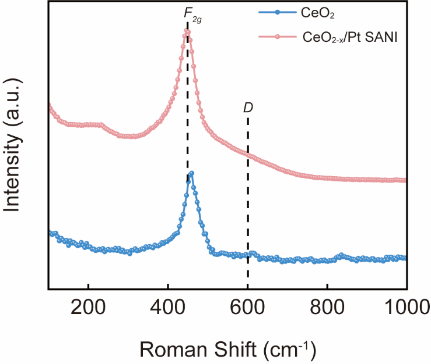


**Fig. S4. The Raman spectra of CeO_2_ and CeO_2-x_/Pt SANI**


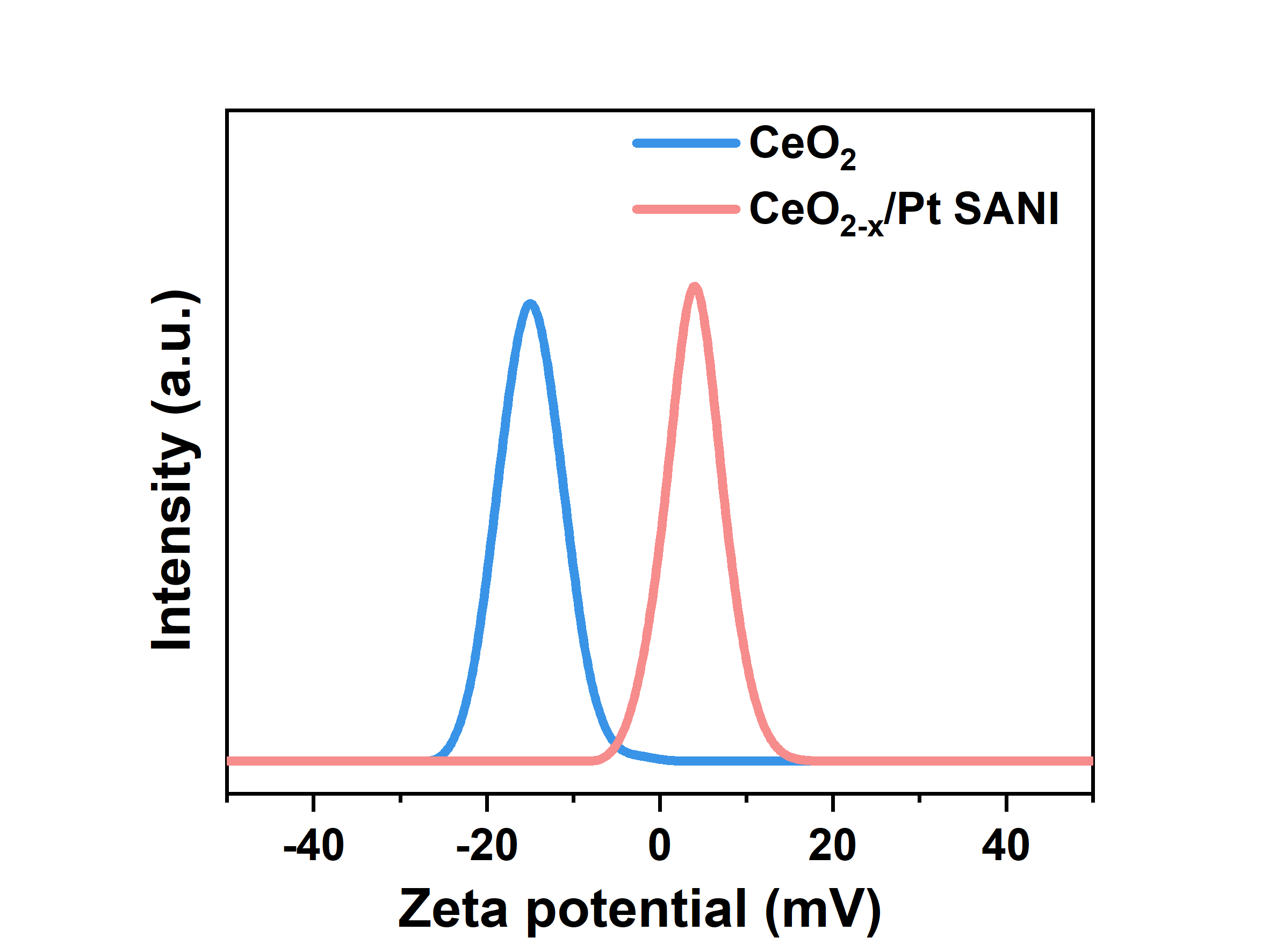


**Fig. S5. Zeta potential distribution of CeO_2_ and CeO_2-x_/Pt SANI.**

**
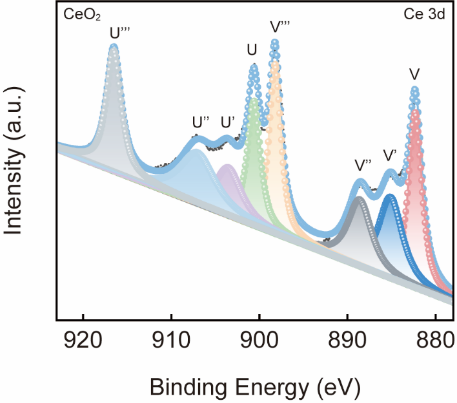
**

**Fig. S6. The high-resolution of Ce 3d in CeO_2_.**


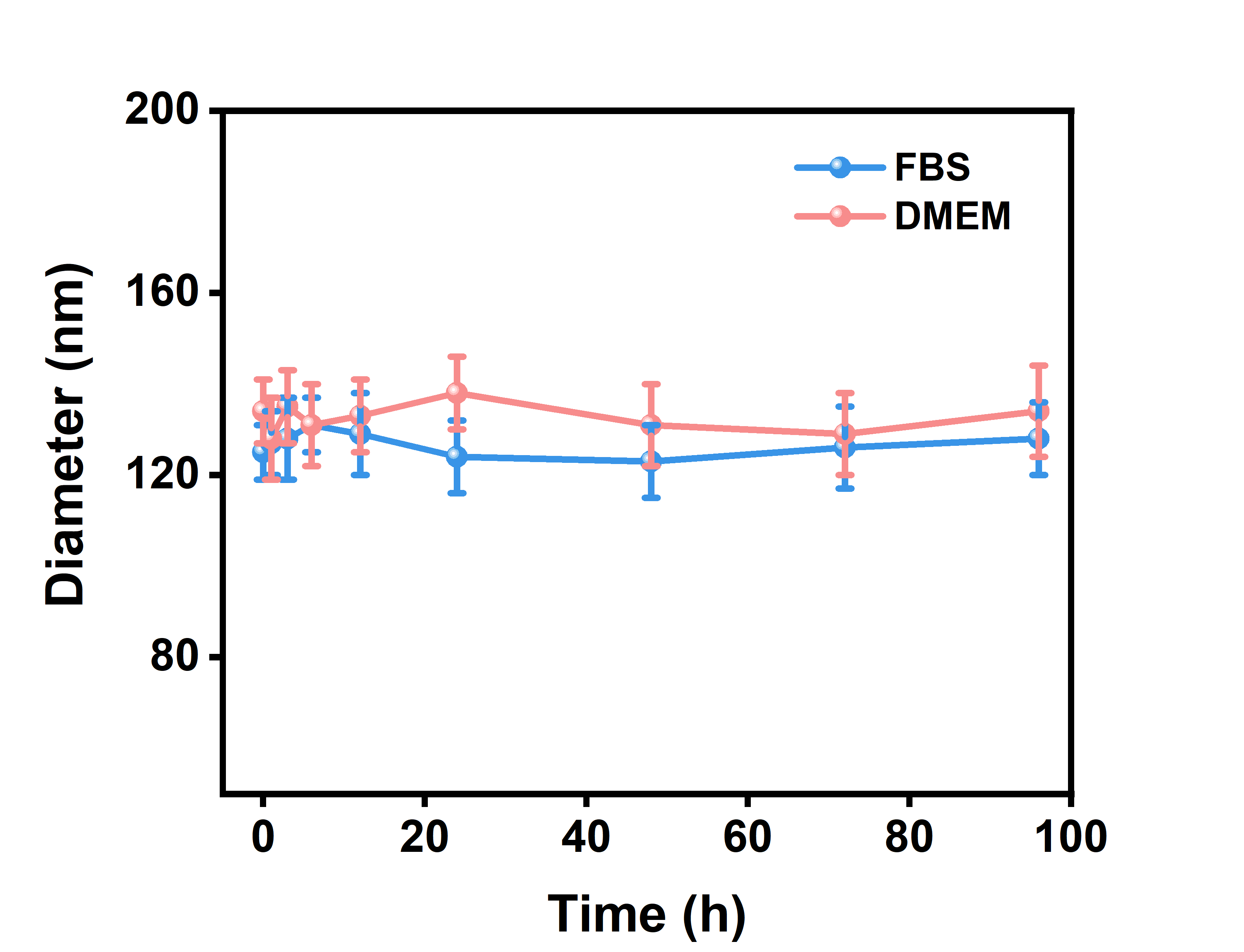


**Fig. S7. Hydrodynamic diameter of CeO_2-x_/Pt SANI incubated in FBS and DMEM over 96 hours. Data are presented as mean ± standard deviation (n=3).**

**
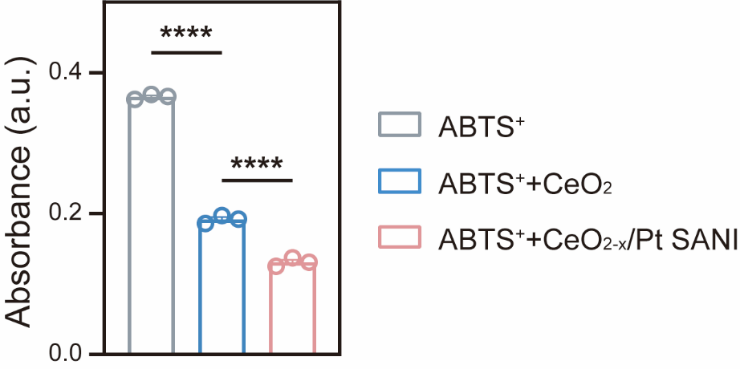
**

**Fig. S8. Bar chart analysis of the ability of CeO_2_ and CeO_2-x_/Pt SANI to eliminate ABTS free radicals.**

**
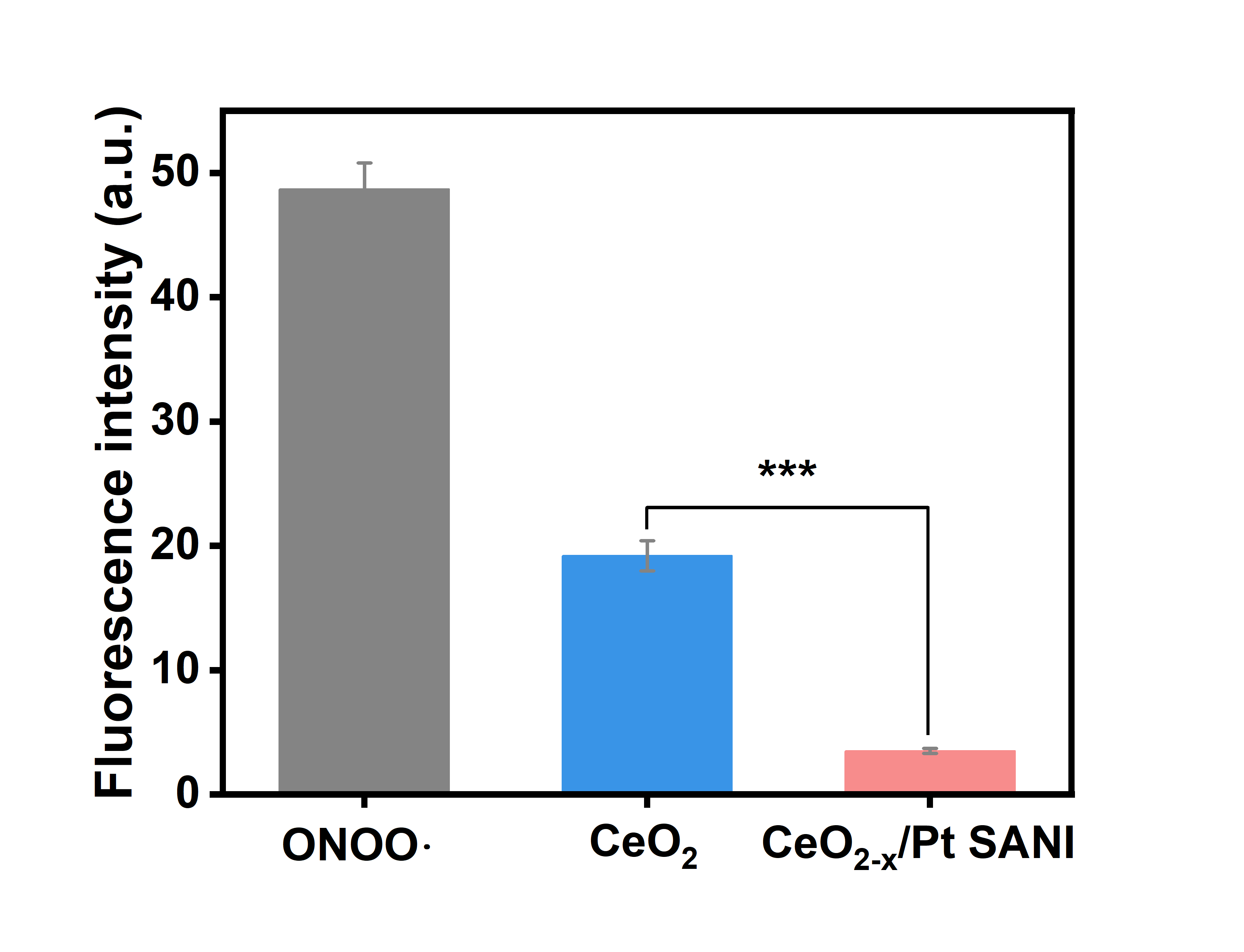
**

**Fig. S9. The RNS scavenging activity of CeO_2_ and CeO_2-x_/Pt SANI using peroxynitrite kit.**

**
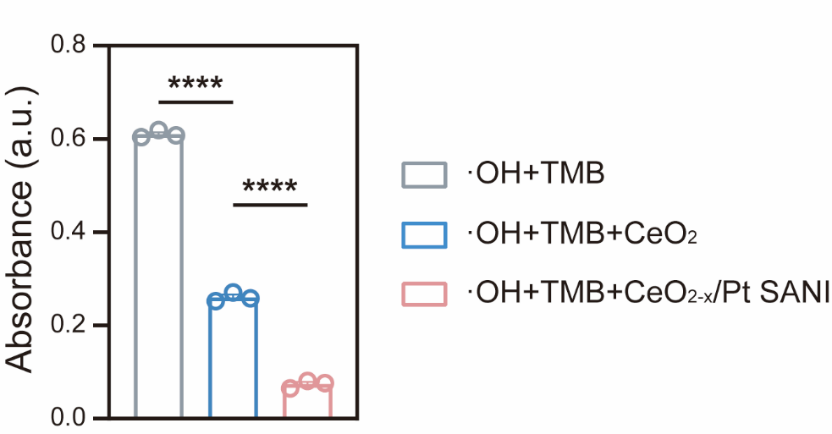
**

**Fig. S10. Bar chart analysis of the ability of CeO_2_ and CeO_2-x_/Pt SANI to eliminate ·OH radicals.**

**
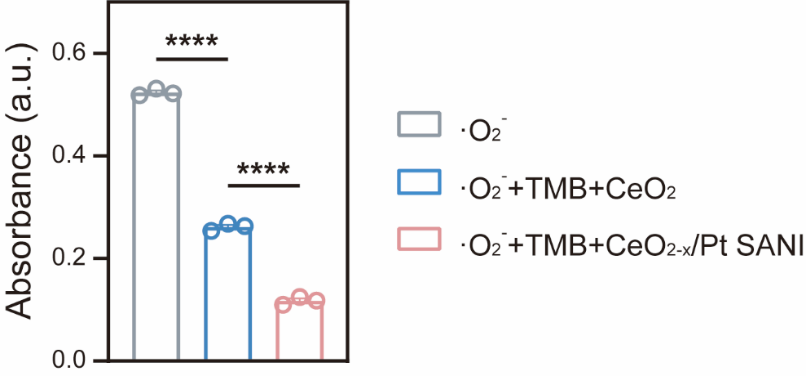
**

**Fig. S11. Bar chart analysis of the ability of CeO_2_ and CeO_2-x_/Pt SANI to eliminate ·O_2_^-^ radicals.**

**
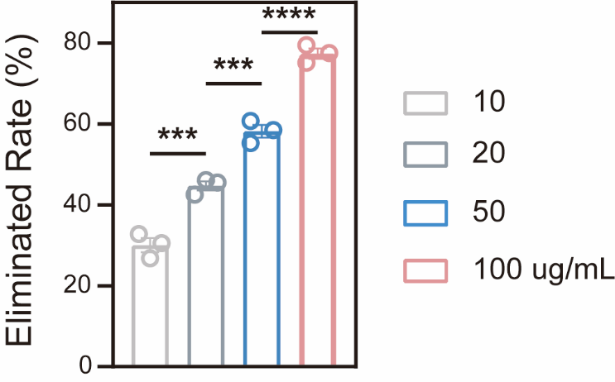
**

**Fig. S12. Bar chart analysis of the ability of CeO_2-x_/Pt SANI to eliminate ·O_2_^-^ radicals at different concentrations.**

**
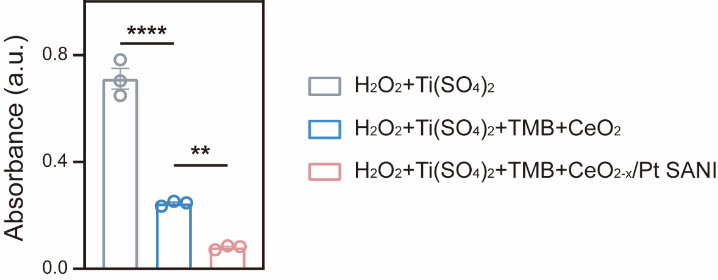
**

**Fig. S13. Bar chart analysis of the ability of CeO_2_ and CeO_2-x_/Pt SANI to eliminate H_2_O_2_.**


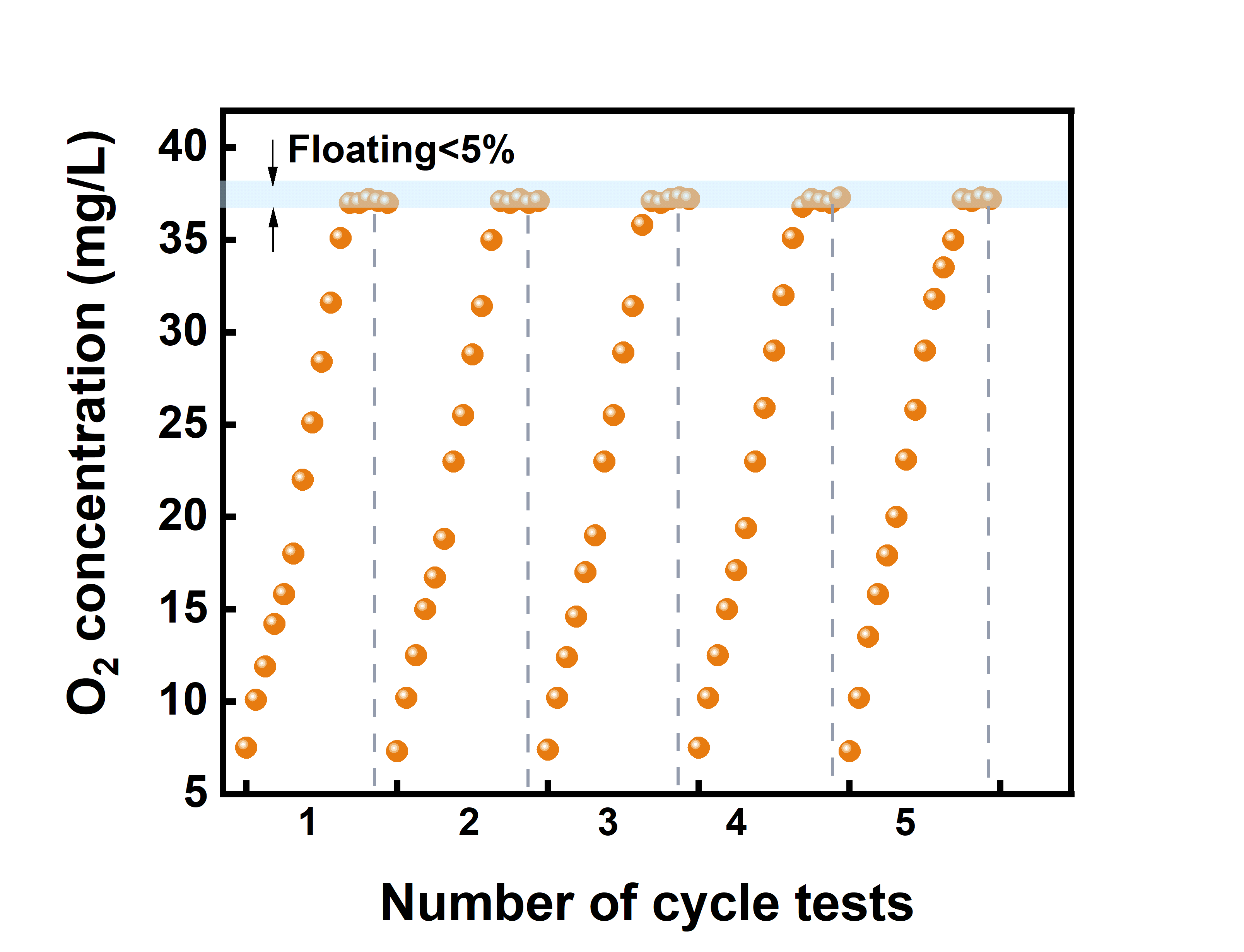


**Fig. S14. Catalytic stability of CeO_2-x_/Pt SANI evaluated by O_2_ generation over multiple cycles.**

**
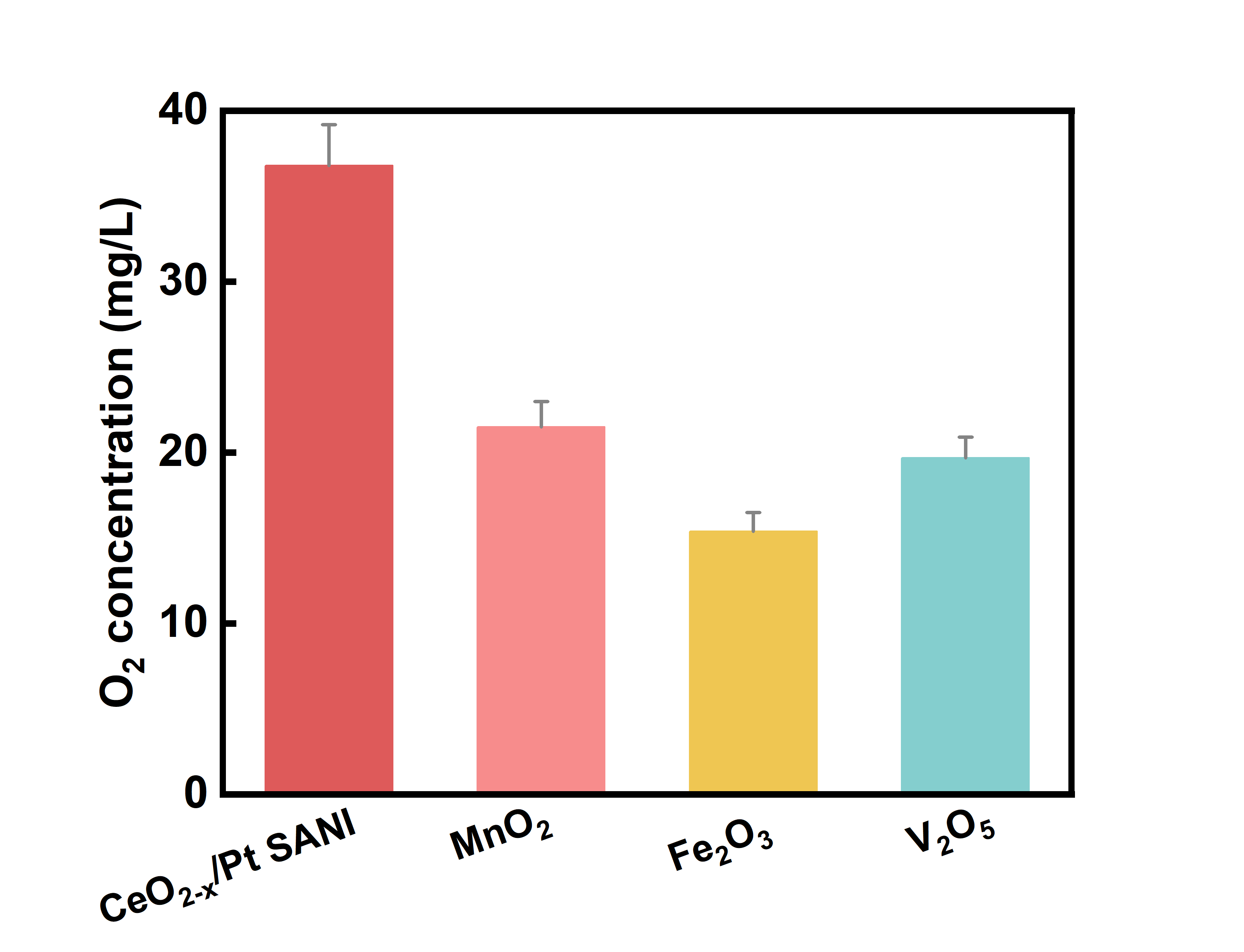
**

**Fig. S15. The CAT-like activity of different nanozymes based on O_2_ concentration.**


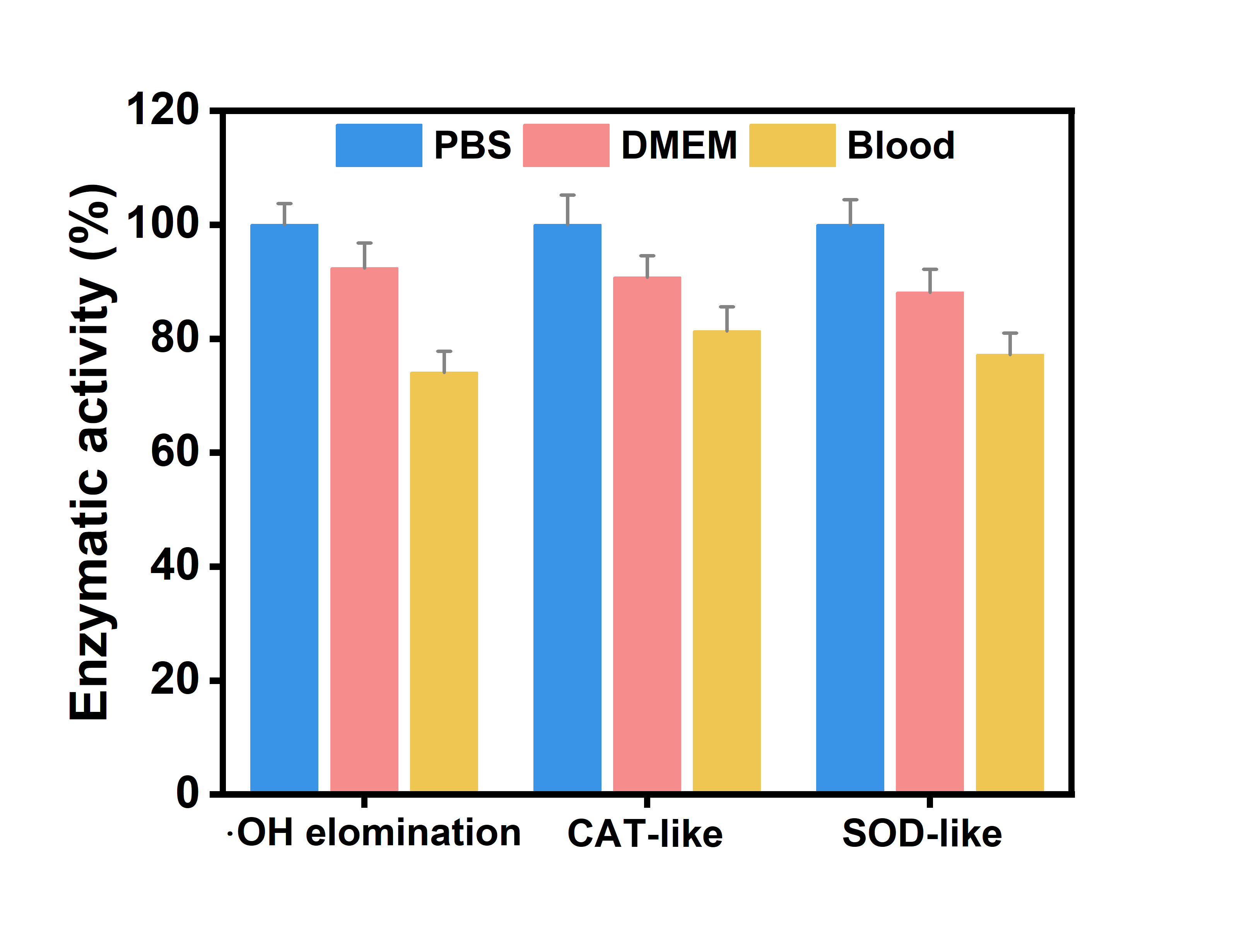


**Fig. S16. ROS-scavenging activity of CeO_2-x_/Pt SANI in different conditions.**

**
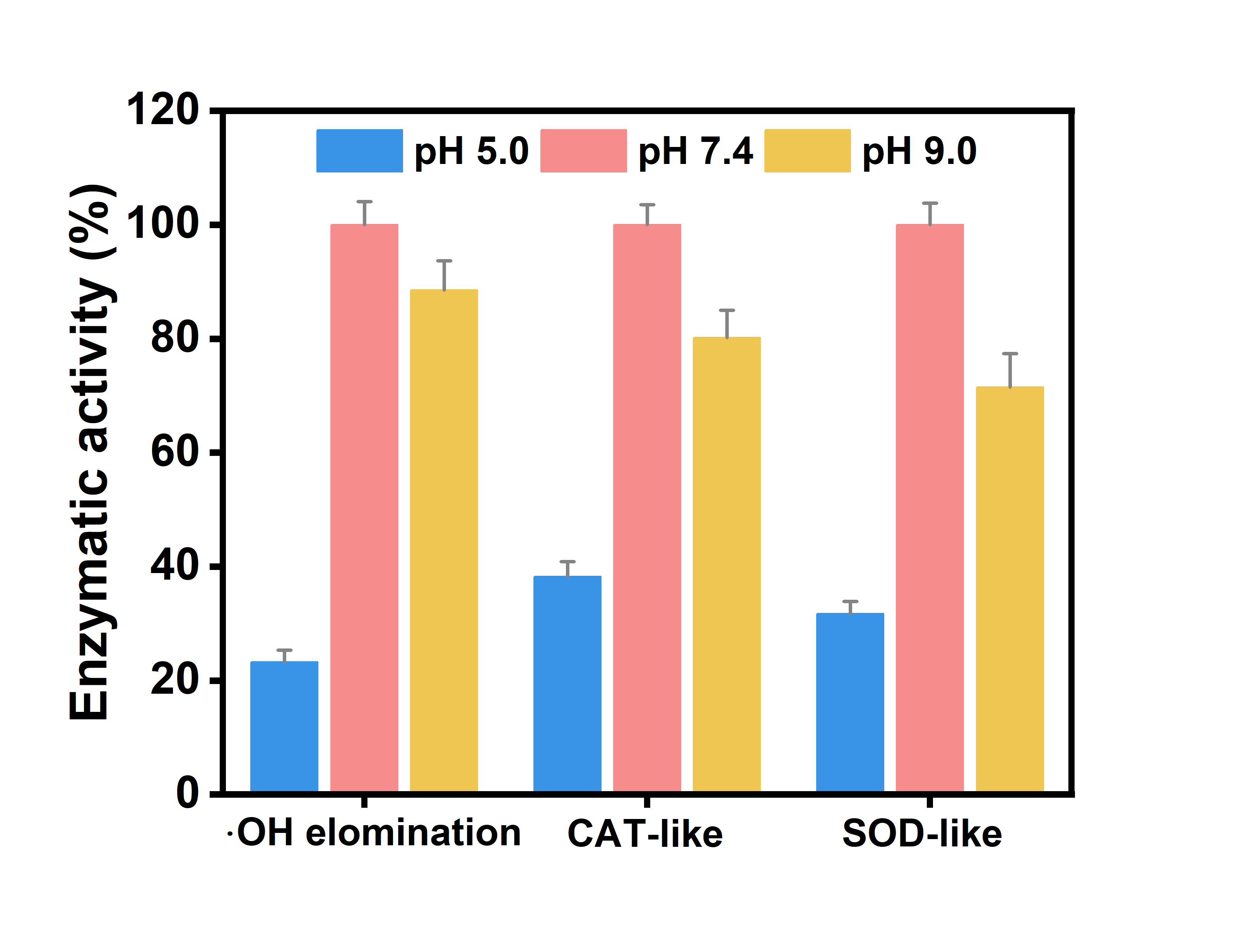
**

**Fig. S17. ROS-scavenging activity of CeO_2-x_/Pt SANI in different pH.**

**
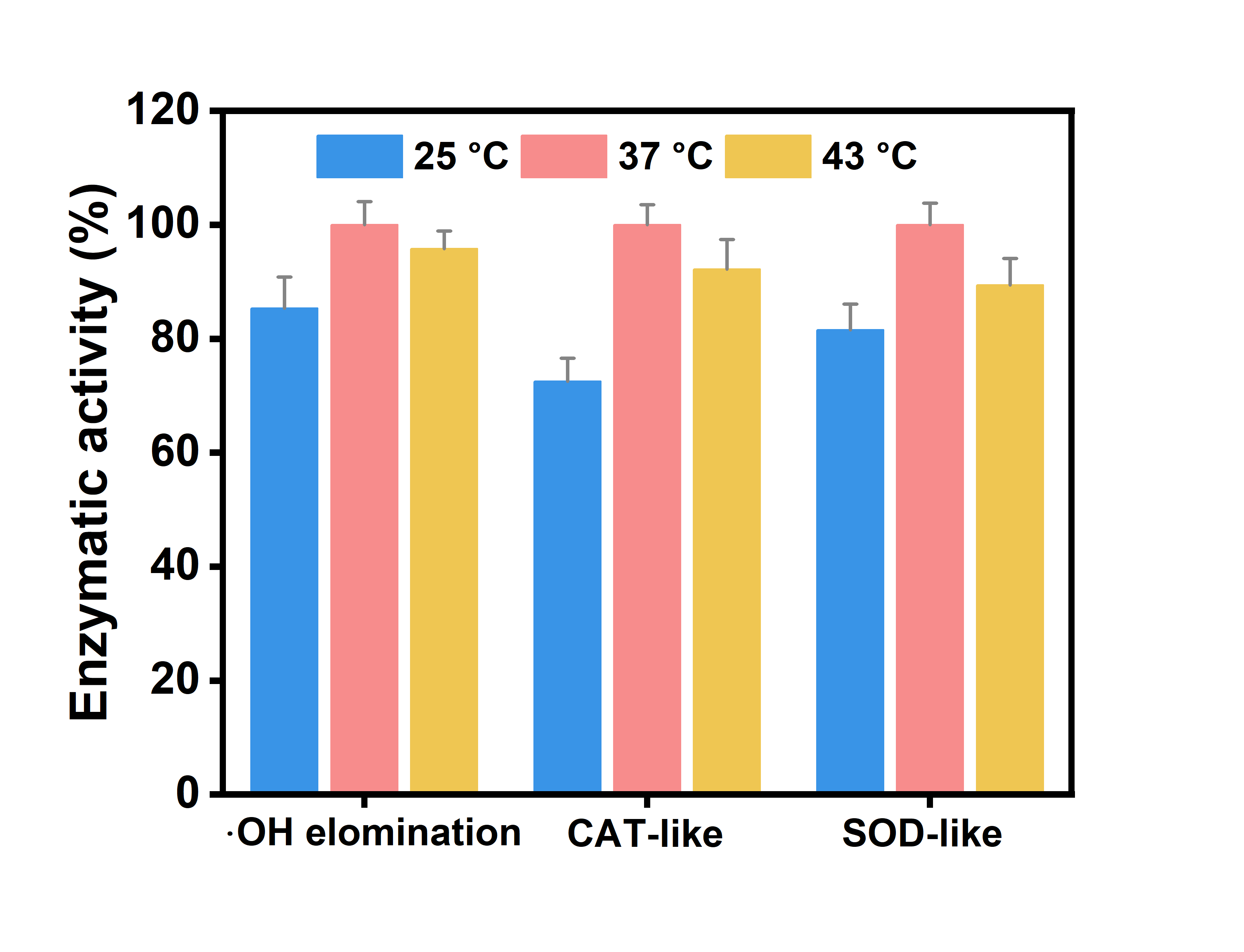
**

**Fig. S18. ROS-scavenging activity of CeO_2-x_/Pt SANI in different temperature.**

**
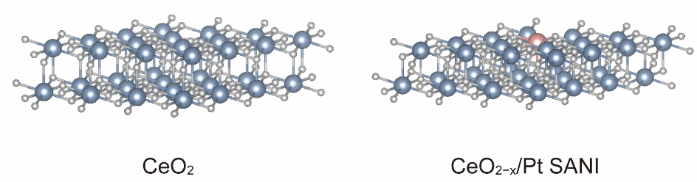
**

**Fig. S19. The geometrically optimized structures of CeO_2-x_/Pt SANI and CeO_2_.**

**
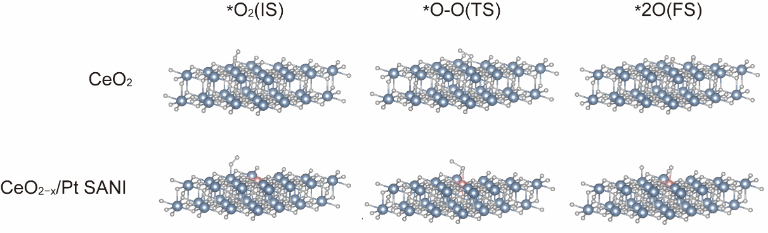
**

**Fig. S20. The** **energy barriers for O_2_ adsorption and dissociation to form 2O in CeO_2-x_/Pt SANI and CeO_2_ structures.**

**
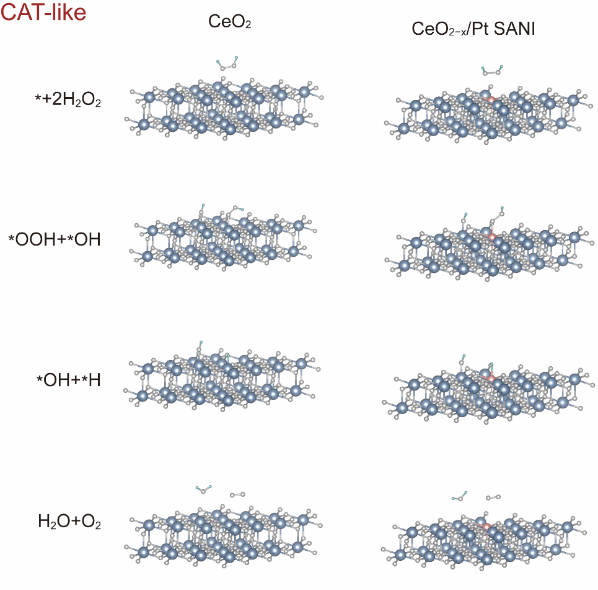
**

**Fig. S21. Gibbs free-energy diagrams for the decomposition of the conversion of H_2_O_2_ into O_2_ on CeO_2-x_/Pt SANI and CeO_2_.**

**
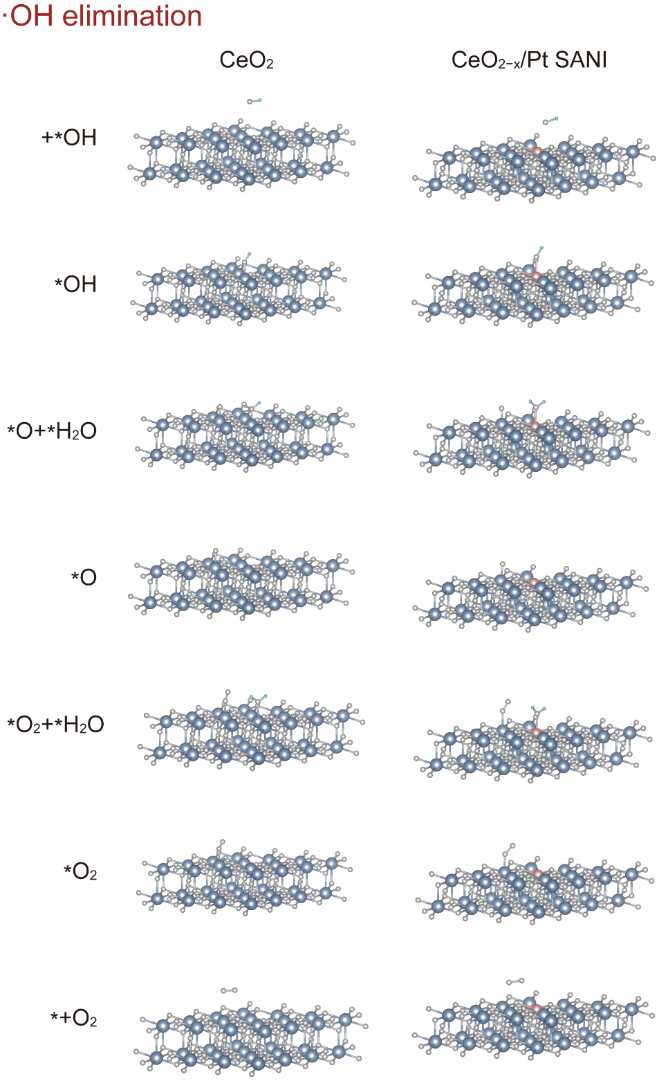
**

**Fig. S22. Gibbs free-energy diagrams for the decomposition of •OH into H_2_O on CeO_2-x_/Pt SANI and CeO_2_.**

**
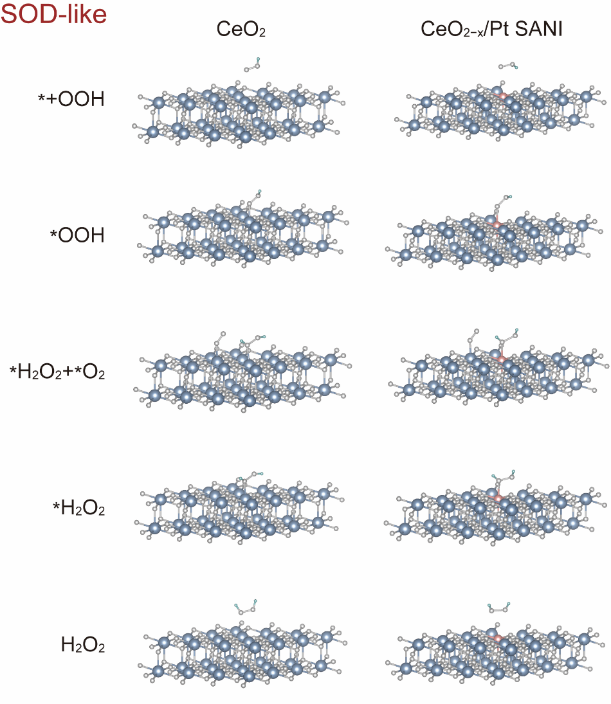
**

**Fig. S23. Gibbs free-energy diagrams for the decomposition of •O_2_^-^ into H_2_O_2_ on CeO_2-x_/Pt SANI and CeO_2_.**

**
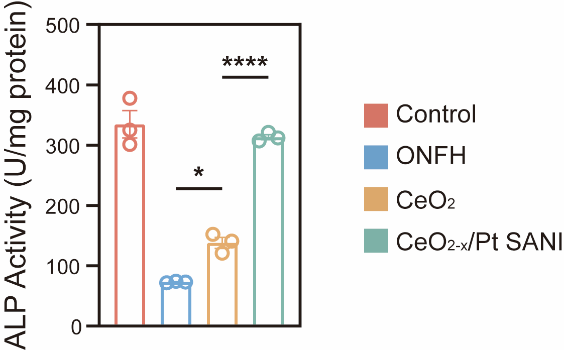
**

**Fig. S24. Quantitative analysis of ALP activity was performed to evaluate osteogenic capacity of HBMSCs under different conditions. Data are presented as mean ± SD. One-way ANOVA with Tukey’s post hoc test was used for multiple comparisons. * P<0.05, **** P<0.0001.**

**
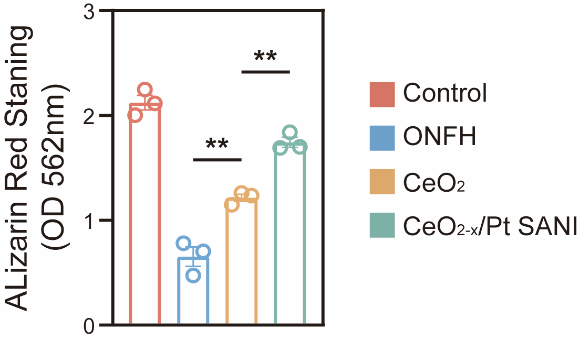
**

**Fig. S25. Quantitative analysis of Alizarin Red staining was performed to evaluate osteogenic capacity of HBMSCs under different conditions. One-way ANOVA with Tukey’s post hoc test was used for multiple comparisons. ** P<0.01.**

**
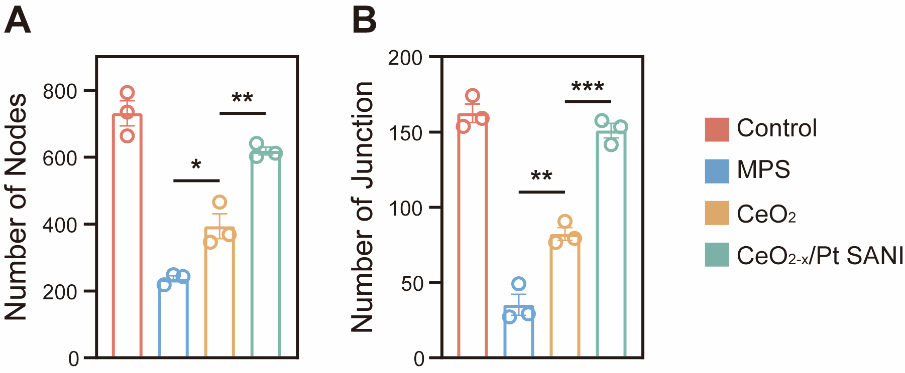
**

**Fig. S26. Network nodes and junctions of HBMSCs under different conditions. Data are presented as mean ± SD. One-way ANOVA with Tukey’s post hoc test was used for multiple comparisons. * P<0.05, ** P<0.01, *** P<0.001.**

**
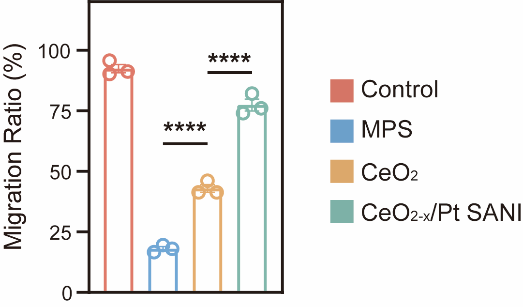
**

**Fig. S27. Migration rate of HBMSCs under different conditions. Data are presented as mean ± SD. One-way ANOVA with Tukey’s post hoc test was used for multiple comparisons. **** P<0.0001.**

**
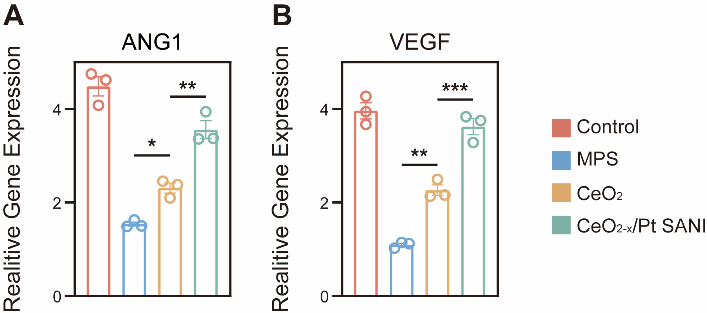
**

**Fig. S28. qRT-PCR analysis of angiogenic factors. Data are presented as mean ± SD. One-way ANOVA with Tukey’s post hoc test was used for multiple comparisons. * P<0.05, ** P<0.01, *** P<0.001.**

**
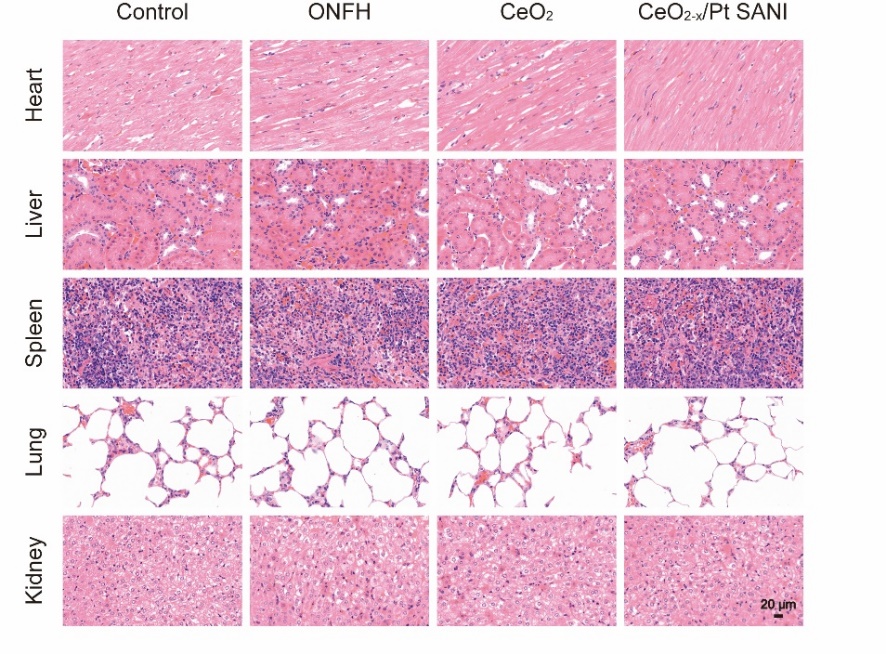
**

**Fig. S29. Representative H&E-stained sections of heart, liver, spleen, lung and kidney tissues from rats in four groups. No signs of inflammation, necrosis, hemorrhage or other pathological alterations were observed in any organ across all treatment groups. Scale bar, 20 µm.**


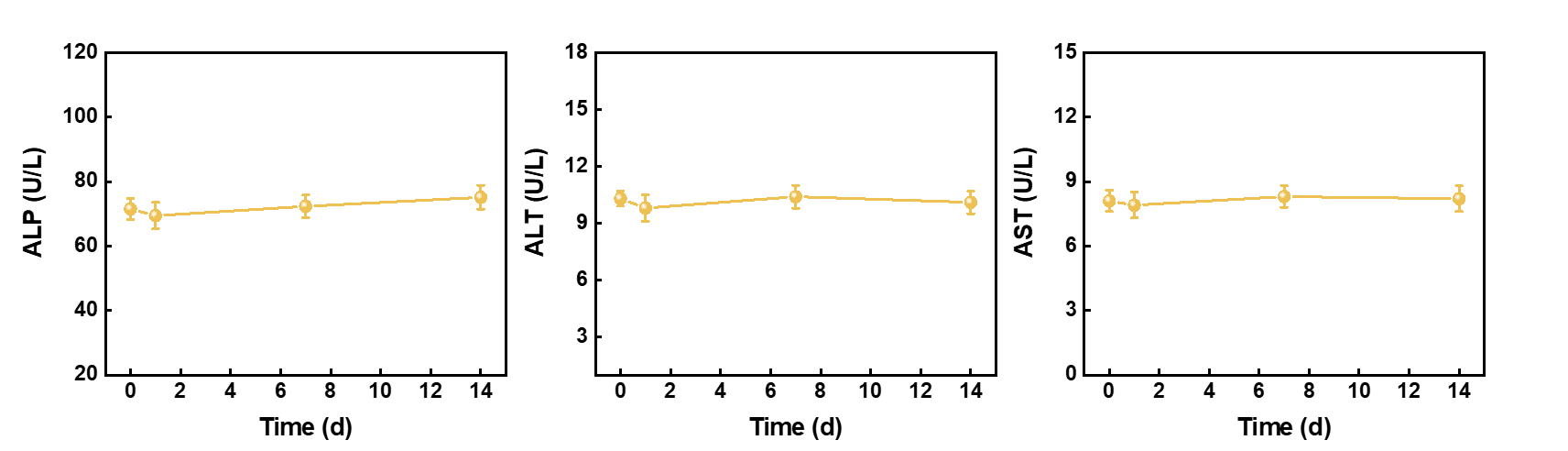


**Fig. S30. Blood biochemistry analysis of mice after injection with CeO_2-x_/Pt SANI at different time points. (a) Alkaline phosphatase (ALP), (b) Alanine transaminase (ALT), and (c) Aspartate aminotransferase (AST) levels at days 1, 7, and 14**.

**
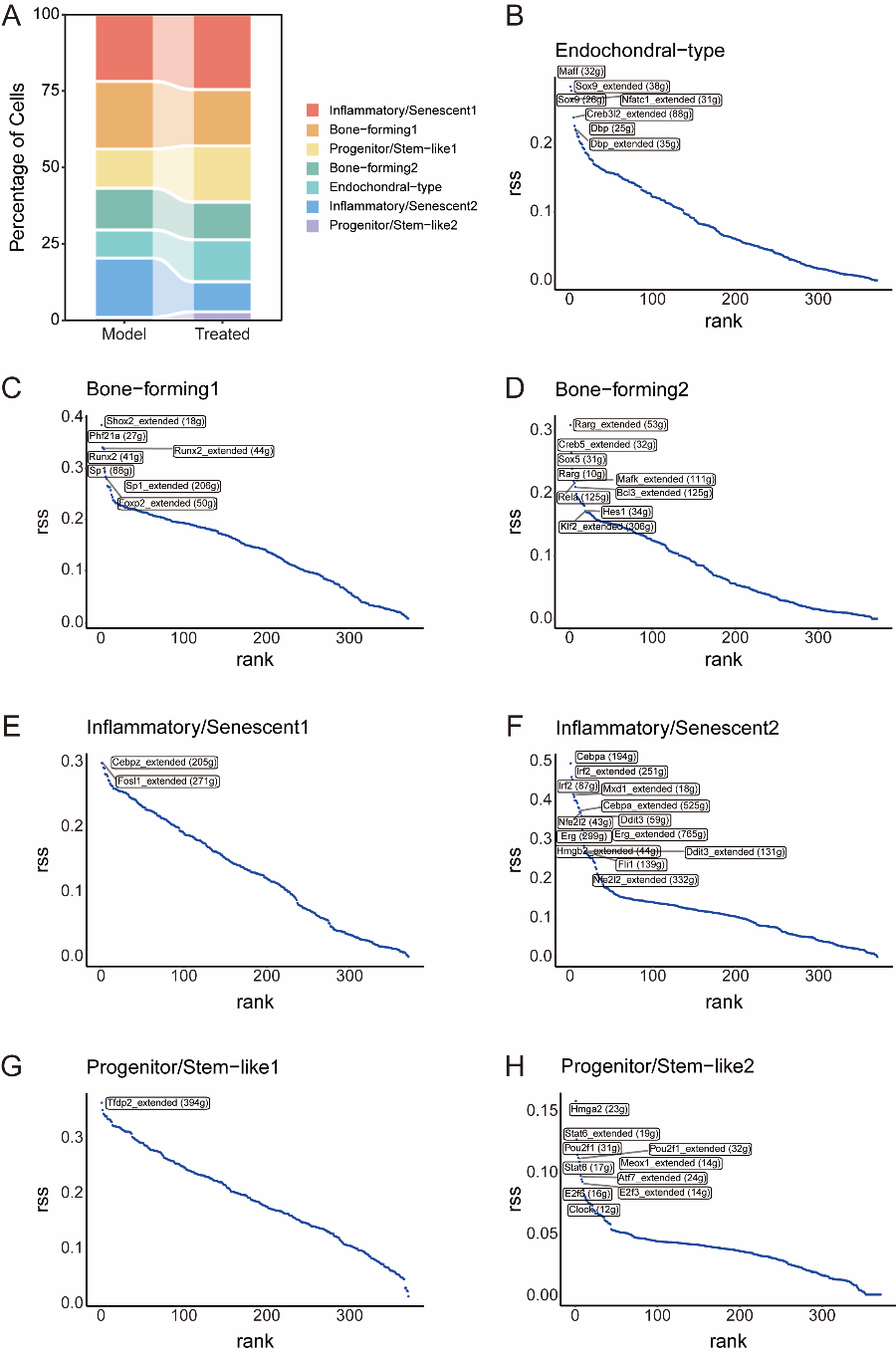
**

**Fig. S31. (A) Proportional distribution of HBMSCs subpopulations between SONFH model (Model) and CeO_2-x_/Pt SANI -treated (Treated) groups, showing a notable shift from senescent/inflammatory clusters to bone-forming and progenitor-like states following treatment. (B-H) Representative regulon specificity scores (RSS) of key transcription factors enriched in each subpopulation, as determined by SCENIC analysis. Top ranked regulons are annotated in each panel.**

**
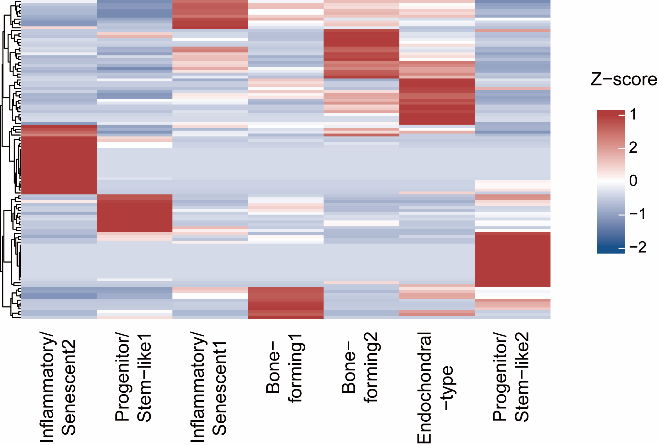
**

**Fig. S32. scRNA-seq reveals HBMSCs subpopulation heterogeneity and lineage trajectory under different conditions. Unsupervised clustering and heatmap visualization of HBMSCs sextracted from integrated scRNA-seq data reveal distinct transcriptional signatures across seven subpopulations.**

**
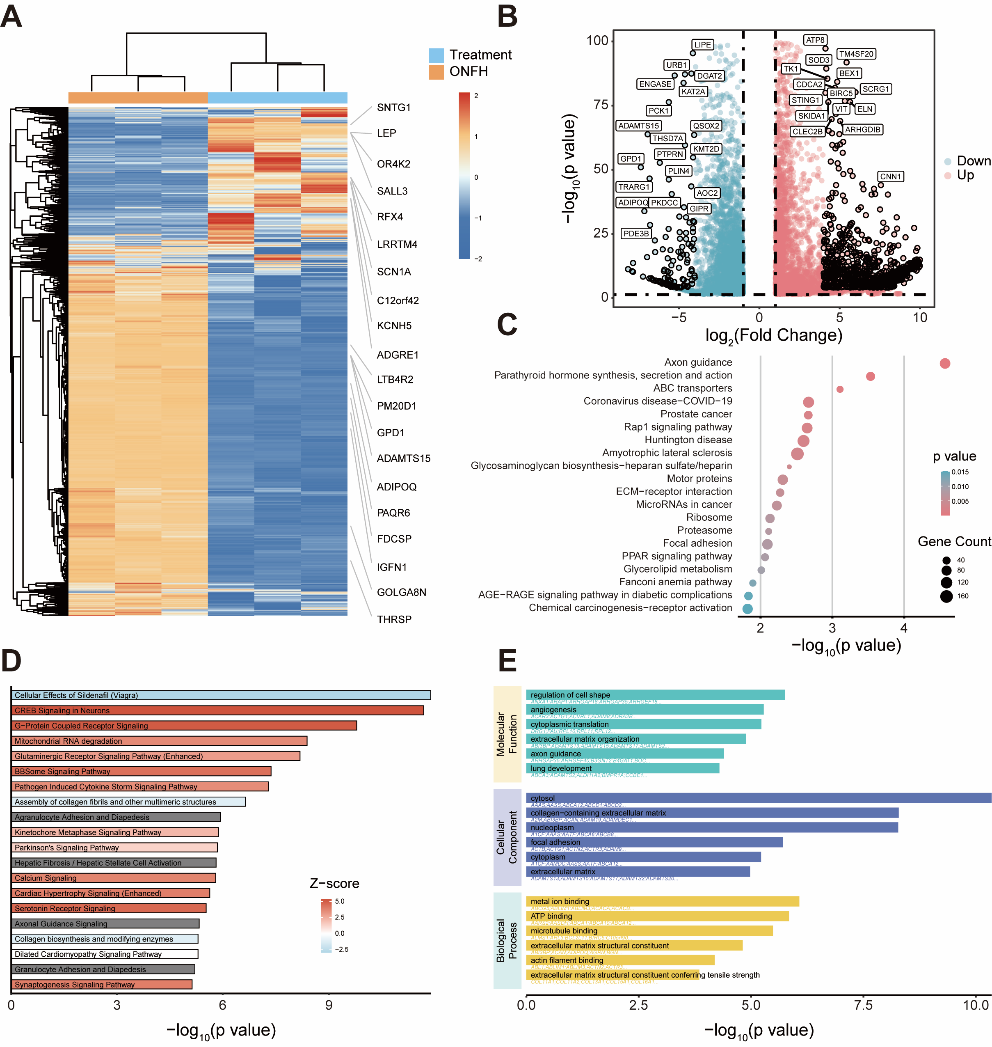
**

**Fig. S33. (A) Hierarchical clustering heatmap of differentially expressed genes (DEGs) between SONFH and** **CeO_2-x_/Pt SANI-treated groups, showing widespread reversal of gene expression patterns upon treatment. (B) Volcano plot highlighting upregulated (red) and downregulated (blue) genes in the treatment group versus ONFH (fold change > 1.5 and p < 0.05). (C) KEGG pathway enrichment analysis of DEGs, showing significant enrichment in pathways such as Rap1, ECM-receptor interaction, and PPAR signaling, which are relevant to angiogenesis and bone repair. (D) IPA canonical pathway analysis visualizing significantly altered pathways based on z-score and p-value. Pathways related to mitochondrial dysfunction, oxidative phosphorylation, and extracellular matrix remodeling were markedly regulated. (E) Gene Ontology (GO) enrichment analysis classifying DEGs by molecular function, cellular component, and biological process. Treatment upregulated genes involved in mitochondrial components, actin cytoskeleton organization, and collagen binding—supporting restored cellular metabolism and structural integrity.**

**
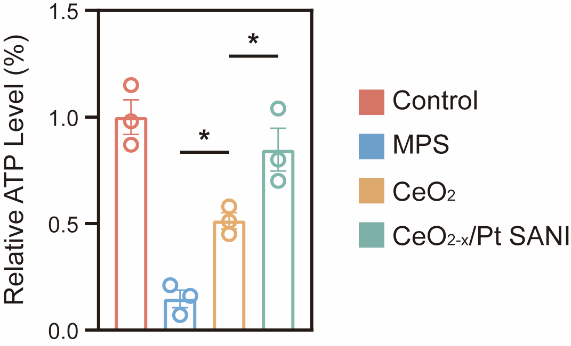
**

**Fig. S34. Quantification of relative ATP levels in femoral head-derived osteoblastic cells from Control, MPS, CeO₂-treated, and CeO_2-x_/Pt SANI-treated groups. Data are presented as mean ± SD. One-way ANOVA with Tukey’s post hoc test was used for multiple comparisons. * P<0.05.**

**Table S1. EXAFS data fitting results of Samples.**

| **Sample** | **Path** | **CN^a^** | **R(Å)^b^** | **σ^2^ (****Å^2^)^c^** | **ΔE_0_(eV)^d^** | **R factor** |
| --- | --- | --- | --- | --- | --- | --- |
| **Pt L_3_-edge (Ѕ_0_^2^=0.878)** | | | | | | |
| **Pt foil** | **Pt-Pt** | **12.0*** | **2.764±0.001** | **0.0048** | **7.1** | **0.0004** |
| **PtO_2_** | **Pt-O** | **6.3±0.3** | **2.026±0.005** | **0.0034** | **11.8** | **0.0092** |
|  | **Pt-Pt** | **6.0±0.2** | **3.089±0.005** | **0.0030** | **6.5** |  |
|  | **Pt-O** | **7.0±1.9** | **3.684±0.017** | **0.0034** | **11.8** |  |
| **CeO_2-x_/Pt SANI** | **Pt-O** | **3.7±0.4** | **1.980±0.007** | **0.0030** | **9.5** | **0.0177** |

*^a^CN*, coordination number; *^b^R*, the distance between absorber and backscatter atoms; *^c^σ*^2^, the Debye Waller factor value; *^d^ΔE*_0_, inner potential correction to account for the difference in the inner potential between the sample and the reference compound; *R* factor indicates the goodness of the fit. *S*0^2^ was fixed to 0.878, according to the experimental EXAFS fit of Pt foil by fixing *CN* as the known crystallographic value. * This value was fixed during EXAFS fitting, based on the known structure of Pt. Fitting conditions: *k* range：3.0 - 9.0; *R* range: 1.0-2.0; fitting space: R space; *k*-weight = 2. A reasonable range of EXAFS fitting parameters: 0.800 < *Ѕ*_0_^2^ < 1.000; *CN >* 0; *σ*^2^ > 0 Å^2^; |Δ*E*_0_| < 15 eV; *R* factor < 0.02.

**
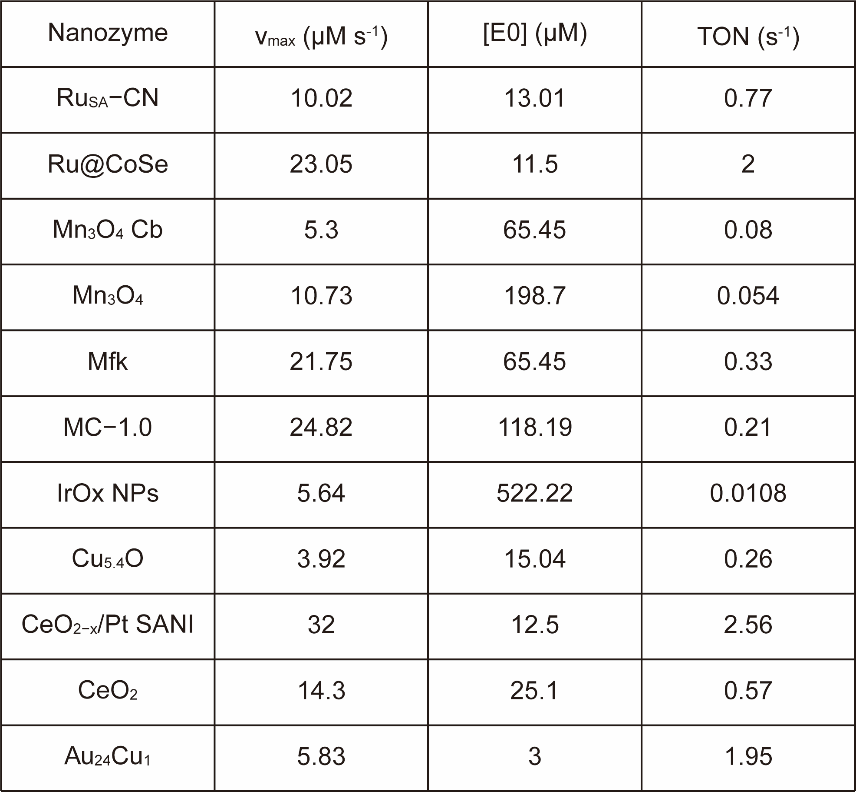
**

**Table S2. Comparison and analysis of the TON, [E_0_], and V_max_ values with previously reported nanozyme.**

**
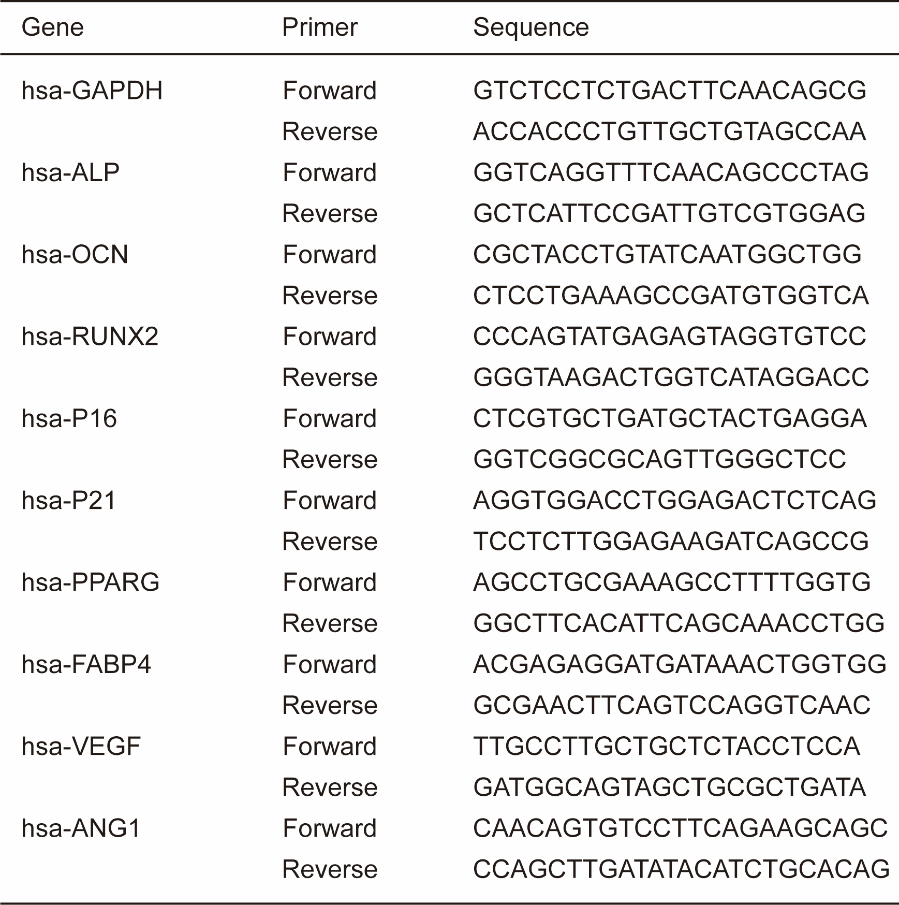
**

**Table S3. Primer sequences used for qRT-PCR in this study.**
